# Bias-Corrected CMIP5 Projections for Climate Change and Assessments of Impact on Malaria in Senegal under the VECTRI Model

**DOI:** 10.3390/tropicalmed8060310

**Published:** 2023-06-06

**Authors:** Papa Fall, Ibrahima Diouf, Abdoulaye Deme, Semou Diouf, Doudou Sene, Benjamin Sultan, Adjoua Moïse Famien, Serge Janicot

**Affiliations:** 1 Laboratoire Environnement-Ingénierie-Télécommunication-Energies Renouvelables (LEITER), Unité de Formation et de Recherche de Sciences Appliquées et de Technologie, Université Gaston Berger de Saint-Louis, BP 234, Saint-Louis 32000, Senegal; 2 Laboratoire de Physique de l’Atmosphère et de l’Océan–Siméon Fongang, Ecole Supérieure Polytechnique de l’Université Cheikh Anta Diop (UCAD), BP 5085, Dakar-Fann, Dakar 10700, Senegal; 3 Programme National de Lutte Contre le Paludisme (PNLP), BP 5085, Dakar-Fann, Dakar 10700, Senegal; 4 ESPACE-DEV, Université Montpellier, IRD, Université Guyane, Université Réunion, Université Antilles, Université Avignon, 34093 Montpellier, France; 5 Laboratoire d’Océanographie et du Climat: Expérimentations et Approches Numériques (LOCEAN), Sorbonne Université, IRD, CNRS, MNHN, 75005 Paris, France; 6 Département de Sciences et Techniques, Université Alassane Ouattara de Bouaké, Bouaké 01 BPV 18, Côte d’Ivoire

**Keywords:** climate change, malaria, Senegal, VECTRI, GCM, CDF-t method, BIAS corrected CMIP5

## Abstract

On the climate-health issue, studies have already attempted to understand the influence of climate change on the transmission of malaria. Extreme weather events such as floods, droughts, or heat waves can alter the course and distribution of malaria. This study aims to understand the impact of future climate change on malaria transmission using, for the first time in Senegal, the ICTP’s community-based vector-borne disease model, TRIeste (VECTRI). This biological model is a dynamic mathematical model for the study of malaria transmission that considers the impact of climate and population variability. A new approach for VECTRI input parameters was also used. A bias correction technique, the cumulative distribution function transform (CDF-t) method, was applied to climate simulations to remove systematic biases in the Coupled Model Intercomparison Project Phase 5 (CMIP5) global climate models (GCMs) that could alter impact predictions. Beforehand, we use reference data for validation such as CPC global unified gauge-based analysis of daily precipitation (CPC for Climate Prediction Center), ERA5-land reanalysis, Climate Hazards InfraRed Precipitation with Station data (CHIRPS), and African Rainfall Climatology 2.0 (ARC2). The results were analyzed for two CMIP5 scenarios for the different time periods: assessment: 1983–2005; near future: 2006–2028; medium term: 2030–2052; and far future: 2077–2099). The validation results show that the models reproduce the annual cycle well. Except for the IPSL-CM5B model, which gives a peak in August, all the other models (ACCESS1–3, CanESM2, CSIRO, CMCC-CM, CMCC-CMS, CNRM-CM5, GFDL-CM3, GFDL-ESM2G, GFDL-ESM2M, inmcm4, and IPSL-CM5B) agree with the validation data on a maximum peak in September with a period of strong transmission in August–October. With spatial variation, the CMIP5 model simulations show more of a difference in the number of malaria cases between the south and the north. Malaria transmission is much higher in the south than in the north. However, the results predicted by the models on the occurrence of malaria by 2100 show differences between the RCP8.5 scenario, considered a high emission scenario, and the RCP4.5 scenario, considered an intermediate mitigation scenario. The CanESM2, CMCC-CM, CMCC-CMS, inmcm4, and IPSL-CM5B models predict decreases with the RCP4.5 scenario. However, ACCESS1–3, CSIRO, NRCM-CM5, GFDL-CM3, GFDL-ESM2G, and GFDL-ESM2M predict increases in malaria under all scenarios (RCP4.5 and RCP8.5). The projected decrease in malaria in the future with these models is much more visible in the RCP8.5 scenario. The results of this study are of paramount importance in the climate-health field. These results will assist in decision-making and will allow for the establishment of preventive surveillance systems for local climate-sensitive diseases, including malaria, in the targeted regions of Senegal.

## 1. Introduction

It is now recognized that vector-borne diseases, including malaria, are largely related to climate variability. Many studies have shown the risk of malaria transmission to be primarily modulated by rainfall, temperature, and humidity [1,2,3]. In Senegal, malaria remains endemic throughout the country. Throughout the country, the entire population is at risk of the disease. It is recognized in Senegal that malaria is a major cause of negative socioeconomic impact, mortality, and morbidity [4]. Between 2017 and 2020, the number of malaria cases decreased by 4.4%, from 52 to 50 per 1000 population at risk, while the number of malaria deaths increased slightly by 1.8%, from 0.24 to 0.245 per 1000 population at risk during the same period. In 2020, 0.7% of malaria deaths worldwide occurred in the country [4].

In Senegal, malaria transmission is closely linked to the rhythm of the rains. It generally occurs during the rainy season and at the beginning of the dry season because of. The main vectors of malaria belong to the *An. gambiae* complex and *An. arabiensis*. These two high-vector-density species belonging to the *An. gambiae* complex are responsible for all malaria transmission [5]. The disease is caused by three Plasmodial species (*Plasmodium falciparum, Plasmodium malaria* and *Plasmodium ovale*) of which the main one is *Plasmodium falciparum* [6,7].

According to the 2007 Intergovernmental Panel on Climate Change (IPCC) report [8], climate change on the Earth’s surface is unequivocal. As a result of its immediate and sustained impact on the natural environment, climate change has become a major threat to the planet. These climatic changes directly threaten human life. These health problems include vector-borne diseases, heat-related disorders, and mental health disorders [9,10]. Many deaths and illnesses are due to climate change and variability through natural disasters such as heat waves, floods, and droughts.

Several studies in Senegal show that climate change is having a major impact on the health sector. Studies have already attempted to understand the effects of climate change on malaria transmission [11,12]. The evolution and distribution of malaria can be affected by extreme weather events, such as floods or droughts. Otherwise, modifying the rainfall system in intensity and frequency can modulate the development of the mosquito population [13,14,15,16].

The important climate simulation exercises carried out by climate modeling teams have greatly contributed to our understanding of the evolution of climate and climate change over the 20th and 21st centuries. This work has made it possible to know the interactions and feedback between its different components (atmosphere, biosphere, cryosphere, oceans, and continental surface), to understand the climate system, and also to understand climate change of anthropogenic origin and its consequences on societies and the environment [17,18]. This is of great interest to scientists and remains a major environmental and scientific challenge. The best tools for simulating climate change are coupled atmosphere–ocean general circulation models (AOGCM) [8,19]. Two main groups of climate models differ according to their resolution: the global circulation models (GCM) [20,21] and the regional circulation models (RCM) [22]. These models are established by mathematical equations solved on three-dimensional grids. These grids represent vertical and horizontal resolutions. Two main parts make up any climate model: a dynamic part, which describes the equations of the general circulation of the atmosphere, and a physical component introduced into the models in the form of physical parameterizations [23]. Climate models provide outputs that will always have characteristics that differ from observations [24]. This is why statistical processes are applied to them to correct the distortion between observations and simulations. These statistical processes are so-called statistical downscaling techniques when the spatial resolution of the original data are smaller than the final resolution of the corrected data [25] and bias correction when the resolution of the original and corrected data remain the same [26,27].

The first objective of this paper is to evaluate the bias-corrected GCM data obtained with the cumulative distribution function (CDF-t) transformation method in Senegal and then quantify the sensitivity of the bias-corrected data for the study of climate impact on malaria. The second aim of this study is to understand the effects of future climate change on malaria transmission using, for the first time in West Africa, the VECtor-borne disease community model of ICTP, TRIeste (VECTRI), which has been developed by the International Centre for Theoretical Physics (ICTP). The model physics and associated parameters are taken from the literature for the *Anopheles gambiae* complex and the malaria parasite *Plasmodium falciparum*. This biological model is a mathematical model of malaria transmission. The model takes into account the impact of climate variability (rainfall and temperature) and variability in the growth cycles of the malaria parasite and vector. In the simulation, the model also takes into account the population density. The VECTRI model is an open-source tool for understanding the drivers of malaria transmission. The simulation can be done on a regional scale at spatial resolutions of 10 km or less. The model has a compartmental representation of susceptibility, exposure, infectivity, and recovery (SEIR). The VECTRI model can integrate migration, immunity, and interventions [28].

## 2. Materials and Methods

### 2.1. Study Area

**Figure 1:**
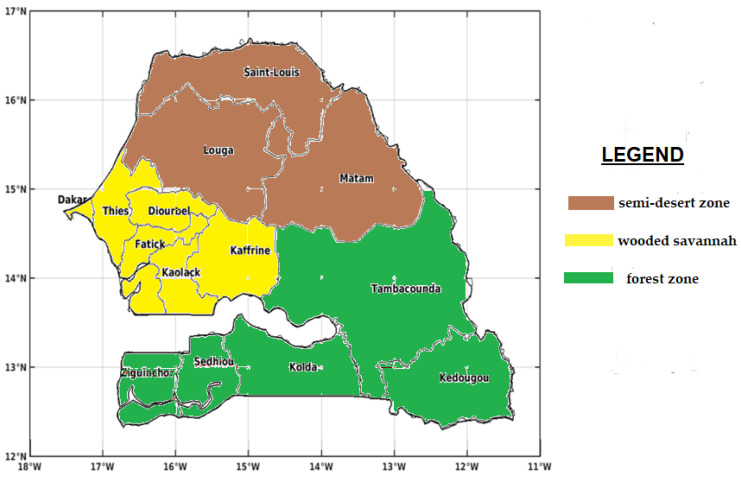
Map of Senegal with its fourteen administrative regions divided according to the agro-climatic zones.

Senegal is a West African country located between the tropics and the equator. It has a tropical climate characterized by the alternation of, on the one hand, the rainy season from July to October and, on the other hand, the dry season from November to June due to the West African Monsoon (WAM) [9]. The annual precipitation varies in a decreasing manner along the south–north direction of the country. In the south, the rains are abundant, reaching up to 1500 mm, while the center–north is part of the Sahel, and receives scanty rains, less than 600 mm per year, with variations from one year to the next. According to the decreasing variation in rainfall, three major rainfall zones corresponding to three climatic zones are identified: a semi-desert zone in the north, the wooded savannah in the center, and a forest zone in the south, with their respective types of climates: a Sahelian climate, a Sudano-Sahelian climate, and a Sudano-Guinean climate [29]. Due to these climatic differences, malaria transmission is unevenly distributed over the territory. Senegal has an area estimated at 196,712 km^2^, divided into 14 administrative regions: Dakar, Diourbel, Fatick, Kaffrine, Kaolack, Kedougou, Kolda, Louga, Matam, Saint-Louis, Sedhiou, Tambacounda, Thies, and Ziguinchor (Figure 1).

### 2.2. Climate Data

For the evaluation part of this study, we use different reference datasets, including re-analysis data (ERA5), estimated satellite data (ARC2 and CHIRPS), and CPC observations from meteorological station networks around the world, and on the other hand, data from different global climate models (GCM) of the CMIP5 project (Coupled Model Intercomparison Project Phase 5 (CMIP5) for the historical data and the projections.

ERA5 data represent the terrestrial component of ERA5 reanalysis climate data from 1979 to the present [30]. The data has a 0.25° × 0.25° (25 km × 25 km) resolution grid, and the temporal frequency of output is hourly [31].The Climate Hazards Group InfraRed Rainfall with Station data (CHIRPS) is a rainfall dataset covering more than 30 years. CHIRPS creates gridded rainfall time series using satellite imagery and in situ station data for resolutions of 0.05° [32]. Africa Rainfall Climatology 2.0 (ARC2) is a 29-year rainfall estimation dataset focused on Africa at a spatial resolution of 0.1°. ARC2 is a revision of the first version of ARC1 that uses data from two sources in accordance with version 2 of the operational precipitation estimation algorithm [33].The Climate Prediction Center (CPC) is a gridded dataset of precipitation and temperature with a resolution of 0.5°. These data have been available since 1979 [28].Three objective techniques are applied to data from daily quality-controlled precipitation reports from approximately 16,000 land stations to obtain daily precipitation analyses [34]. Table 1 summarizes the re-analysis and data satellite combined with observation data (model, definition, grid, and references).

### 2.3. GCM Models Used (Bias-Corrected CMIP5)

The results of climate impact studies may be sensitive to global climate model (GCM) biases. In the simulation of current precipitation, the biases will impact the reproduction of future precipitation changes. In fact, precipitation changes associated with warming correlate with the present-day pattern of precipitation [35].

Precipitation biases, for example, alter and modify hydrological simulations because of the nonlinear nature of runoff [36]. In addition, temperature biases can influence the distribution of precipitation either as snow or as rain. For this reason, climate impact models generally use bias-corrected GCM output. Researchers working on the evaluation and modeling of climate change impacts (in terms of health, renewable energy, crop yields, water resources, etc.) are increasingly using these simulation outputs either to compute related impact metrics or to alter models.

It is well known that GCM (general circulation model) simulations require a downscaling process to increase their spatial resolution before projecting onto the local domain.

In this study, we extracted the dataset from the daily data produced by [26] using the CDF-t (cumulative distribution function transform) method developed by [37] to correct the statistical distribution of the key climatic parameters for impact simulations.

This bias technique reduces the errors in the present-day simulations of the CMIP5 models compared to observed data according to [12] (figure not shown) and also reduces the spread between models for the different representative concentration pathways (RCPs).

As GCM prediction outputs have a coarse spatial resolution and are not suitable for use directly on the local scale, they have been bi-linearly interpolated onto a 0.5° linear grid before bias correction. This unbiased set of daily rainfall and temperature from 11 CMIP5 models is used under two RCPs (RCP4.5 and RCP8.5) over the period 2006–2100 with the resolution of 0.5° × 0.5°.

The RCP4.5 is a scenario, corresponding to a CO_2_ atmospheric concentration of approximately 650 ppm by 2100, that stabilizes radiative forcing at 4.5 W/m^2^ without ever exceeding that value and leads to an elevation of the global surface temperature between 1.1 °C and 2.6 °C.

Conversely, the RCP8.5 is the high-emission scenario with a high greenhouse gas concentration increase in CO_2_ atmospheric concentration >1000 ppm in 2100 that delivers global warming at an average of 8.5 W/m^2^ across the planet with a temperature increase of about 4.3 °C [38,39].

More details on the CDF-t method can be found in [40,41,42]. The validation of CMIP5 bias-corrected data for Africa, used in this work, can be found in [26]. More details on the names, institutes, resolutions and references of the 11 global climate models used in this study can be found in Table 2.

### 2.4. Malaria Impact Model Used (VECTRI)

The malaria model used is a distributed dynamic model called the VECtor-borne disease community model of ICTP, TRIeste (VECTRI). VECTRI is a new dynamic model of community malaria developed by the Abdus Salam International Centre for Theoretical Physics (ICTP). To simulate this, the model takes into account the influences of temperature and rainfall on the life cycles of parasites and vectors [53,54]. The VECTRI model combines a biological model for vector and parasite life cycles. The disease progression in the human host is given by the susceptible-infectious-recovered compartmental representation (SEIR) [55].

The growth of the immature stages (egg, larvae, and pupae) and the gonotrophic and sporogonic cycles were explicitly solved in the model using a set of cells for each process. This is like the method used with the Liverpool Malaria Model (LMM) [56].

The model is able to reproduce the reduction in entomological inoculation rates (EIR) and prevalence (PR) according to increasing population density, which has been widely observed in Africa [57], although the relationship is too close and strong in the model [58]. The model can be run at regional or continental scales. It has a spatial resolution of 5 to 10 km with a daily time step and considers sub-seasonal climate variations. The model has a structure that facilitates future development to integrate migration, immunity, and interventions. More details on the VECTRI model can be found in [28].

### 2.5. Methods

First of all, as shown by our earlier study over Senegal at a local and national scale [12], we employed the VECTRI model to simulate malaria indices and validated them with malaria surveillance data from the National Malaria control Programme (Programme National de Lutte contre le Paludisme, PNLP) in Senegal and with reference climate products including ERA5, CHIRPS, ARC2 and CPC. The comparison of the spatio-temporal representation between the EIR (model data) and the observed cases showed that VECTRI well simulates malaria transmission in Senegal. Indeed, the simulation of the annual cycle was well reproduced by the model, which gives a difference of 1 month between the peak of rainfall and that of EIR. In addition, the year-to-year variation of the EIR variable and the number of malaria cases showed a coincidence of both high and low transmission years associated with high and low rainfall and temperature, respectively. The spatial representation of the EIR variable showed that the southern zone is the most affected by malaria compared to the northern zone, as was found with the PNLP observation data. As for the simulation of malaria seasonality, the simulation results are almost identical to the surveillance data. The results of this previous validation study allowed us to consider VECTRI as a tool for simulating climate-modulated malaria transmission [12].

Afterwards, this paper is undertaken further in the framework of impact studies with bias-corrected data from the CMIP5 coupled model intercomparison project. It aims to reproduce historical malaria patterns in Senegal and quantify the projected changes under two RCPs, namely RCP.45 and RCP8.5. In this study, we again used climate data from the ERA5, CHIRPS, ARC2, and CPC as reference data for the baseline.

Some previous analyses of the performance of bias-corrected CMIP5 data to represent some climate characteristics revealed an improvement of the corrected products compared to the observations [26,59]. So, the bias-corrected products can be used to generate future projections and in climate change impact studies [60]. In this study, we use the climate data from the bias-corrected GCMs to develop malaria indices in various climatological periods, namely the baseline, i.e., the validation period (1983–2005), historical (1983–2005), the near future (2006–2035), the middle future (2036–2066), and the far future (2066–2095) using historical experiments and RCP4.5 and RCP8.5 scenarios.

The VECTRI model is forced by the daily rainfall and daily temperature of the bias-corrected GCM model, e.g., 11 bias-corrected CMIP5, for the RCP4.5 and RCP8.5 emission scenarios separately. The VECTRI model uses climate data at this native resolution. All the models used were interpolated to the resolution of the Senegal mask, which is 0.0625° longitude for 0.0625° latitude, to standardize the resolutions, improve intercomparison, and achieve the multi-model average. For validation, the time series and spatial patterns of the GCM models are compared to those of the reference datasets, considering the common period. The Taylor diagram described by [61] is used to evaluate the performance of bias-corrected CMIP5 models against reference datasets. It is used by meteorologists and atmospheric scientists. This diagram offers the advantage of representing at the same time 3 statistics: the centered root-mean-square error (RMSE), the standard deviation (STD) of the simulation compared to the observation, and the correlation coefficient between observation and simulation. The correlation coefficient measures the degree of connection or dependence between two quantitative traits. It is between −1 and 1. The standard deviation is the most commonly used measure of data dispersion in statistics. The lower the standard deviation, the less the values are dispersed around their mean. The RMSE of the arithmetic mean of the square of the difference between the observed and simulated values.

A new approach for the VECTRI model input parameters was used. The bias correction technique using the CDF-t (cumulative distribution function transform) method was applied to the climate simulations of the fifth phase of the Coupled Models Intercomparison Project (CMIP5) [26]. This eliminates systematic biases in climate models that could alter impact predictions. The results were analyzed for two representative concentration pathway (RCP) scenarios (RCP4.5 and RCP8.5) for the different periods (historical: 1983–2005; near future: 2006–2035; the medium term: 2036–2065; and far future: 2066–2095).

## 3. Results

### 3.1. Validation of the Rainfall and Temperature Inputs

**Figure 2:**
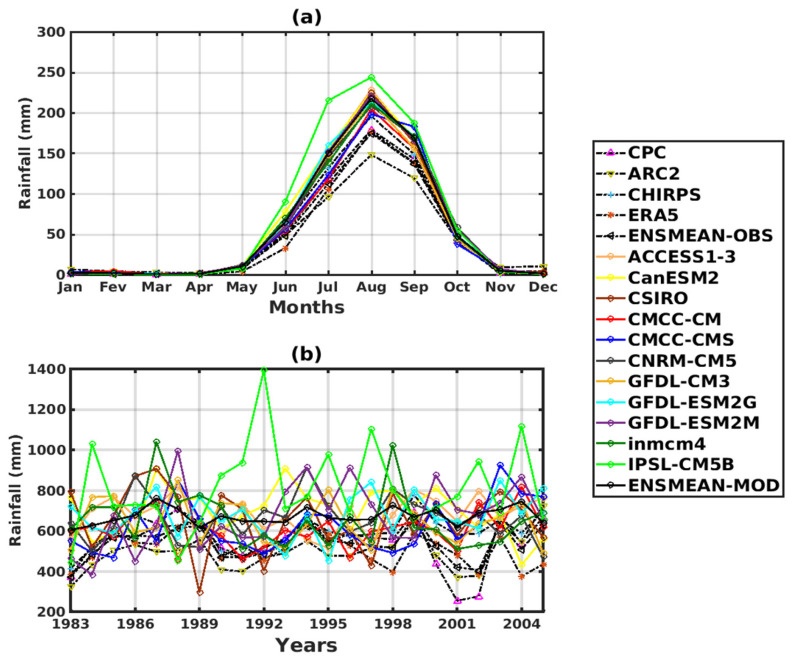
Temporal variability of rainfall for validation data: (the reference data used as observation data) CPC, ARC2, CHIRPS, ERA5, and ENSMEAN-OBS. Corrected GCM data: ACCESS1–3, CanESM2, CSIRO, CMCC-CM, CMCC-CMS, CNRM-CM5, GFDL-CM3, GFDL-ESM2G, GFDL-ESM2M, inmcm4, IPSL-CM5B, ENSMEAN-MOD from 1983 to 2005 in Senegal: (**a**) Annual cycle, (**b**) Interannual cycle.

For the annual cycle (Figure 2a), the datasets show a peak (maximum precipitation) in August. On the other hand, CHIRPS data shows the highest rainfall amount approaching 200 mm in August, while ARC2 shows the lowest rainfall amount in August (~150 mm). A small variation between data sets is observed in the amplitudes.

The CMIP5 climate simulations were compared to these datasets. The annual cycle is well reproduced by the models (Figure 2a), with the maximum precipitation in August, but with some divergences in the amplitudes. Some models have a maximum value of around 200 mm (CMCC-CM and CMCC-CMS) approaching validation data (CHIRPS), others between 205 and 245 mm (ACCESS1–3, CanESM2, CSIRO, CNRM-CM5, GFDL-CM3, GFDL-ESM2G, GFDL-ESM2M, inmcm4, ENSMEAN-GCM), and one of about 250 mm (IPSL-CM5B), which overestimate rainfall over the period 1983–200.

Greater variability prevails in the interannual cycle (Figure 2b) of precipitation. In Figure 2b, all datasets show an increasing trend from 1983 to 1989 (CPC [400–700 mm], ARC2 [310–550 mm], CHIRPS [410–650 mm], ERA5 [400–650 mm], and ENSMEAN [399–640 mm]). Beyond this year, we see that precipitation decreased slightly between 1990 and 1998. The years 2001 and 2002 was marked by a sharp drop in precipitation, which was mainly manifested by the CPC.

The CMIP5 data, for the year-to-year variation, shows that the IPSL-CM5B model strongly overestimates precipitation over almost the entire period compared to the validation datasets. With the exception of a few models such as CSIRO, inmcm4, and GFDL-ESM2M, which slightly overestimate rainfall for some years, all other models, such as ACCESS1–3, CanESM2, CMCC-CM, CMCC-CMS, CNRM-CM5, GFDL- CM3, GFDL-ESM2G, and ENSMEAN-GCM, are close to validation.

**Figure 3:**
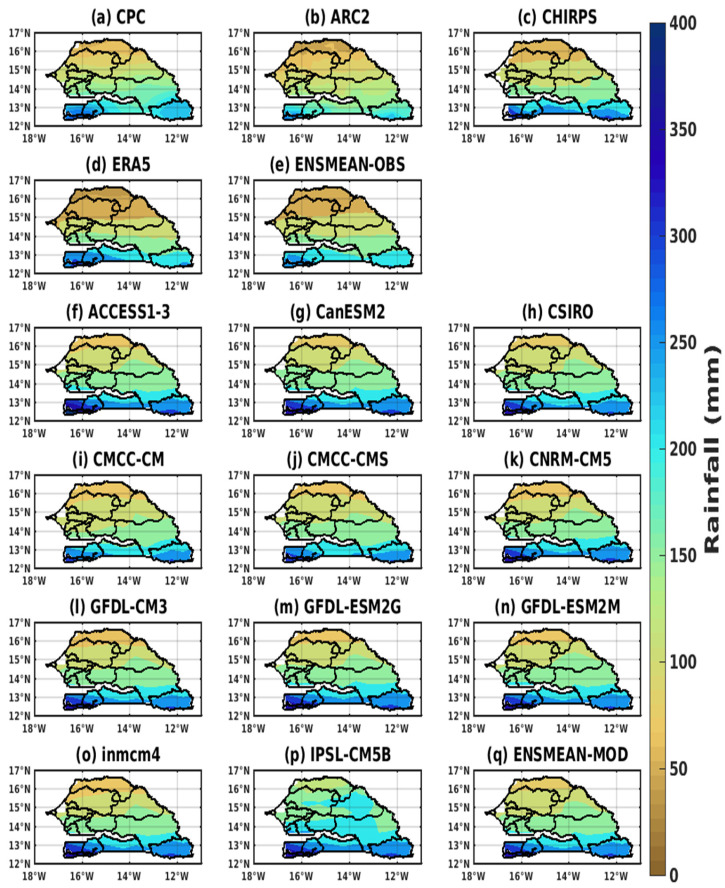
Representation of spatial distribution of rainfall in June, August, and September in Senegal for the common period 1983–2005 for validation data (the reference data used as observation data): (**a**) CPC, (**b**) ARC2, (**c**) CHIRPS, (**d**) ERA5, (**e**) ENSMEAN-OBS, and corrected GCM data: (**f**) ACCESS1–3, (**g**) CanESM2, (**h**) CSIRO, (**i**) CMCC-CM, (**j**) CMCC-CMS, (**k**) CNRM-CM5, (**l**) GFDL-CM3, (**m**) GFDL-ESM2G, (**n**) GFDL-ESM2M, (**o**) inmcm4, (**p**) IPSL-CM5B, and (**q**) ENSMEAN-MOD.

The spatial distribution of precipitation for the different datasets was represented (Figure 3) considering the high rainy season, namely July–September, for the validation period 1983–2005. We recall that there is only one rainy season in Senegal and more generally in the Sahel, between June and October, but that more than 85% of total rainfall falls between July and September (JAS). Indeed, the climate of Senegal presents a unimodal rainfall regime.

The long dry season (seven months) extends from November to May, and the wet or rainy season (five months) from June to October. The maximum rainfall is usually recorded in August. In Figure 3a–e, a noticeable latitudinal gradient separates the northern and southern parts of Senegal. The wettest area of Senegal is clearly highlighted by the different datasets. The regions of Ziguinchor, Sedhiou, Kolda, and Kedougou are located, respectively, in the southwest and southeast of the country.

The spatial pattern of the models is shown in Figure 3f–q for average rainfall in July–August, as this is a period of highest rainfall, usually followed by an upsurge in malaria epidemics. Figure 3f–q illustrate the rainfall gradient from the south to the north of Senegal. The GCM precipitation patterns show a minimum in the central and northern parts, with maximums in the south and southeast, as obtained with the baseline data. The models show the strong signal obtained in the southwestern and southeastern parts as obtained with the validation data (CPC, ARC2, CHIRPS, ERA5, and the ensemble mean).

**Figure 4:**
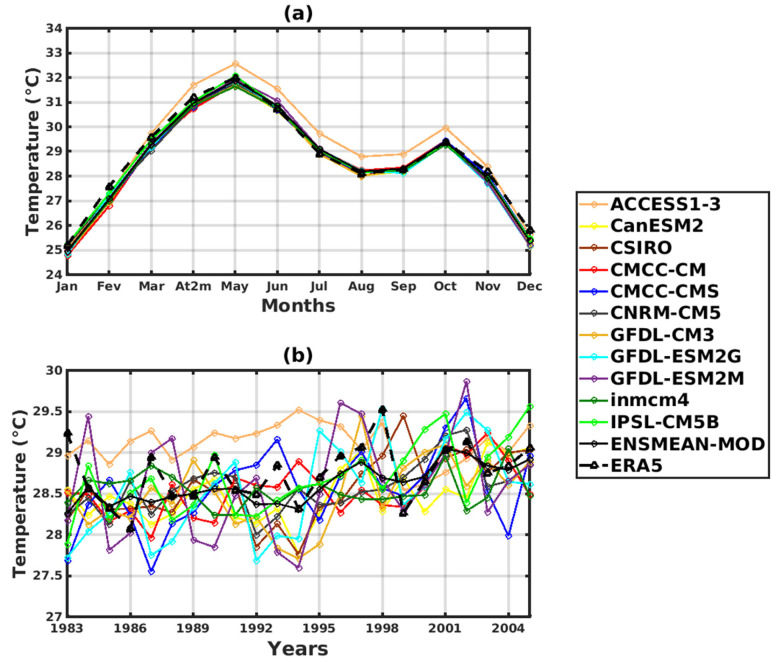
Temporal variability of temperature in Senegal for the period 1983–2005 for validation data (the reference data used as observation data): ERA5. Corrected GCM data: ACCESS1–3, CanESM2, CSIRO, CMCC-CM, CMCC-CMS, CNRM-CM5, GFDL-CM3, GFDL-ESM2G, GFDL-ESM2M, inmcm4, IPSL-CM5B, ENSMEAN-GCM. For historical: (**a**) Annual cycle and (**b**) Interannual cycle.

The annual cycle indicates a bimodal temperature cycle with two peaks in May (31.5 °C) and October (29 °C) (Figure 4a). The monthly temperature trend shows that mild temperature conditions prevail from July to September due to the influence of cloud cover and heavy rainfall during the rainy season. A regular trend resulting in an increase in temperatures is observed. In addition, we even see this tendency to increase in temperature in the validation period, which worsens in the projections.

The annual temperature cycles obtained with the various CMIP5 simulation data clearly reproduce the two peaks in the temperature evolution: the first in May and the second in October. The ACCESS1–3 model (Figure 4a) strongly overestimates the temperatures (32.5 °C in May and 30 °C in October) compared to the other models, which are close to the values of the validation data (31.5 °C in May and 29 °C in October).

For interannual variability (Figure 4b), significant temperature changes are clearly observed. We can observe a regular trend that has resulted in an increase in temperatures since the beginning of the validation period (1983). The year 1986 records the lowest temperature value (27.7 °C), and the year 1998 records the highest temperature value (29.2 °C).

**Figure 5:**
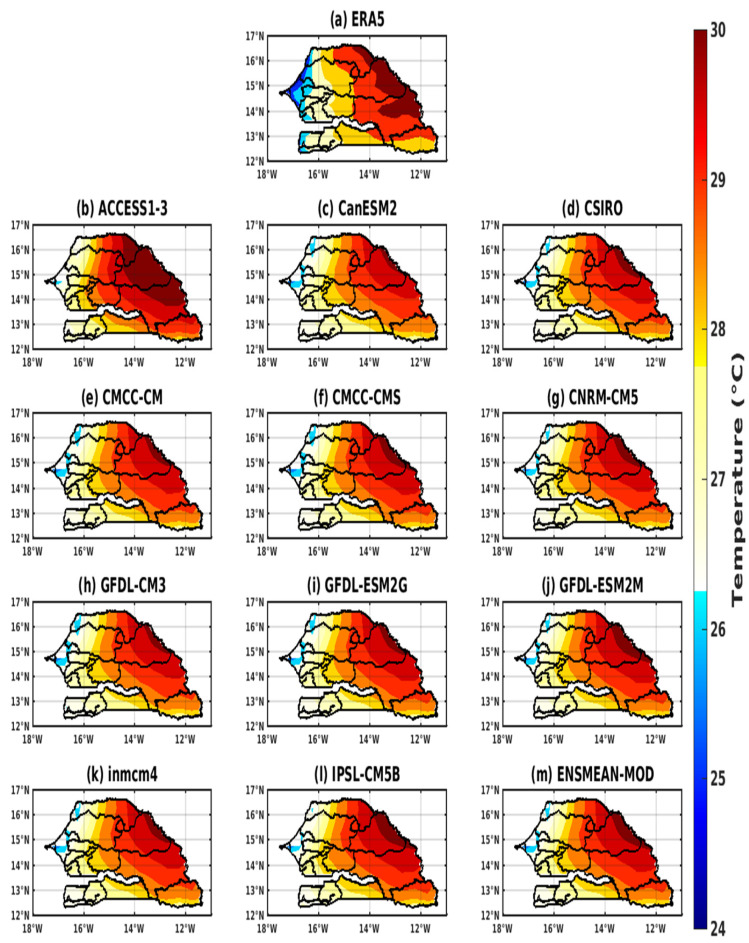
Representation of spatial distribution of temperature for the period 1983–2005 in Senegal for validation data (the reference data used as observation): (**a**) ERA5; corrected GCM data: (**b**) ACCESS1–3, (**c**) CanESM2, (**d**) CSIRO, (**e**) CMCC-CM, (**f**) CMCC-CMS, (**g**) CNRM-CM5, (**h**) GFDL-CM3, (**i**) GFDL-ESM2G, (**j**) GFDL-ESM2M, (**k**) inmcm4, (**l**) IPSL-CM5B, and (**m**) ENSMEAN-MOD.

The spatial distribution reveals that the highest temperatures are generally in the interior of the country but mainly in the eastern part of Senegal, namely in Matam, Tambacounda, and Kédougou. The lowest temperature values prevail in the coastal zone of Senegal (Saint-Louis, Dakar, and Ziguinchor) (Figure 5a). On the coasts, there are winds coming from the ocean that soften the local temperatures, which become low compared to the interior of the territory [62].

The models, for their part, for the spatial distribution show an increasing variation in temperatures from the coast towards the interior of the country (Figure 5b–m). The highest temperatures are recorded in the east of the country. The ACCESS1–3 model overestimates temperatures almost over the spatial extent of the country, although it underestimates the maximum in eastern Senegal and overestimates temperatures on the coast (Figure 5b). Most CMIP5 models indicate an underestimate of the spatial extent of temperatures in the central part of the country. On the other hand, temperatures are overestimated on the coast (Figure 5c–m).

### 3.2. Evaluation of the Performance of the VECTRI Model with GCM

In the following, the results are presented only for the validation of the CMIP5 data to reduce the number of figures. The rest of the figures have been presented in the Supplementary Material.

#### 3.2.1. Spatio-Temporal Variability of the EIR for the Validation of the GCM

**Figure 6:**
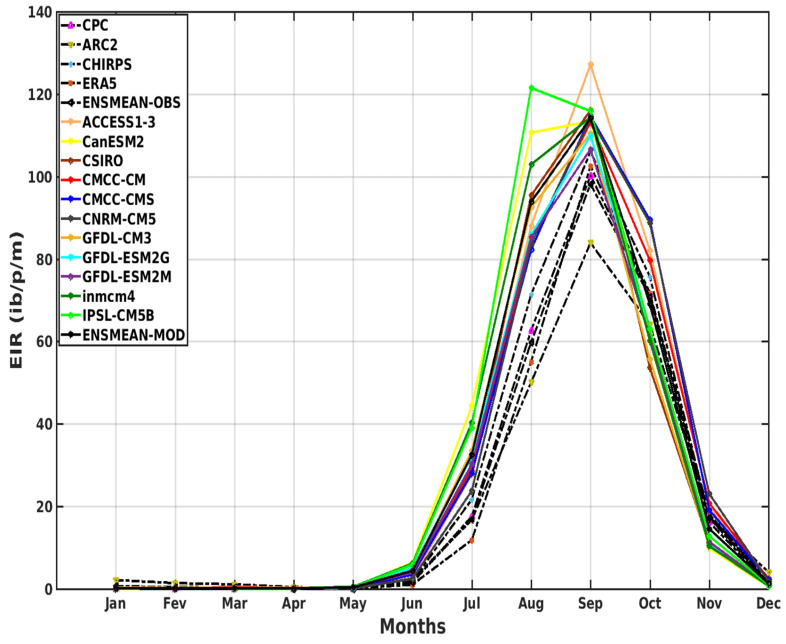
The annual EIR (in ib/p/m, i.e., infectious bites per person per month) cycle of malaria for the period 1983–2005 in Senegal: Simulations of the VECTRI model forced by rainfall and temperature of the CPC, ARC2, CHIRPS, ERA5, and ENSMEAN-OBS (the reference data used as observation data) for evaluation and corrected CMIP5 GCM models from ACCESS1–3, CanESM2, CSIRO, CMCC-CM, CMCC-CMS, CNRM-CM5, GFDL-CM3, GFDL-ESM2G, GFDL-ESM2M, inmcm4, IPSL-CM5B, and ENSMEAN-GCM for historical.

The simulations of the VECTRI model, represented on Figure 6, are based on the reference data and their overall average (CPC, ARC2, CHIRPS, ERA5, and ENSMEAN-OBS) and on the outputs of 11 models from the CMIP5 and their overall mean (ACCESS1–3, CanESM2, CSIRO, CMCC-CM, CMCC-CMS, CNRM-CM5, GFDL-CM3, GFDL-ESM2G, GFDL-ESM2M, inmcm4, IPSL-CM5B, and ENSMEAN-MOD).

The malaria season simulated by VECTRI through the variable EIR (entomological inoculation rate) in Senegal, in particular, runs from August to October, with a sharp peak in September (Figure 6). The highest EIR value is given by CHIRPS (105 ib/p/m), followed by CPC, ERA5, and ENSMEAN-OBS (105 ib/p/m), and finally, ARC2, which has the smallest value (85 lb/p/m).

For the simulation of the CMIPS models, we find that the models reproduced the annual cycle well. Apart from the IPSL-CM5B model, which gives a peak in August (maximum peak at 121 ib/p/m), all the others agree with the validation data over the period with a strong transmission in August, September, and October and a maximum peak in September. All models overestimate malaria in Senegal compared to baseline data. However, the ACCESS1 model greatly exceeds the other models (maximum peak at 125 ib/p/m), followed by the CanESM2, CSIRO, CMCC-CM and CMCC-CMS, CNRM-CM5, inmcm4, and ENSMEAN models (maximum peak at 118 ib/p/m), and finally the GFDL-CM3, GFDL-ESM2G, and GFDL-ESM2M models (maximum peak at 110 ib/p/m).

We note, for the variation of the transmission of malaria during the year according to the latitudes, that the regions located between 13° N and 15° N have a strong tendency for the transmission of malaria during the month of September, where the maximum is observed for validation data (CPC, ARC2, CHIRPS, ERA5, and ENSMEAN-OBS). Above 15° N, the trend gradually decreases at higher latitudes. Regarding the representation of the variation of malaria transmission during the year according to the latitudes of the VECTRI simulations based on the outputs of the CMIP5 models forced by the 11 models and their ensemble mean (ACCESS1–3, CanESM2, CSIRO, CMCC-CM, CMCC-CMS, CNRM-CM5, GFDL-CM3, GFDL-ESM2G, GFDL-ESM2M, inmcm4, IPSL-CM5B, and ENSMEAN-GCM) over the period 1983–2005, the high values of the EIR are found in southern areas at latitudes below 13° N, which gradually decrease or even disappear as they move north, i.e., at latitudes above 17° N. All models (except GFDL-ESM2M and IPSL) have their maximum peak in the month of September, and the period of high malaria transmission is given in August, September, and October (see Figure S1 in the Supplementary Material).

**Figure 7:**
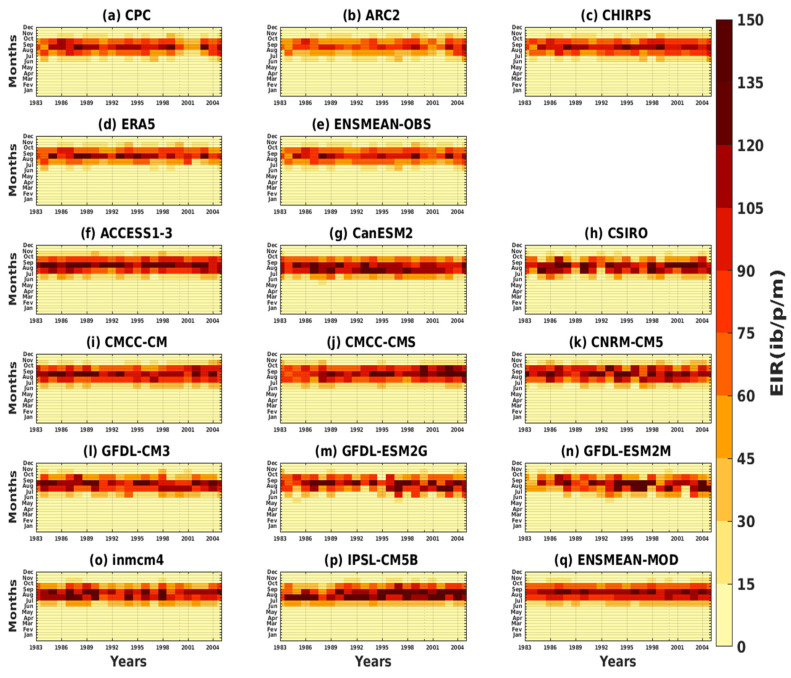
Hovmöller diagram intra- and interannual of the EIR (in ib/p/m, i.e., infectious bites per person per month) of malaria in Senegal for the period 1983–2005. Simulations of the VECTRI model forced by rainfall and temperature of the assessment data: (**a**) CPC, (**b**) ARC2, (**c**) CHIRPS, (**d**) ERA5, and (**e**) ENSMEAN-OBS (the reference data used as observation) for validation and corrected CMIP5 GCM models: (**f**) ACCESS1–3, (**g**) CanESM2, (**h**) CSIRO, (**i**) CMCC-CM, (**j**) CMCC-CMS, (**k**) CNRM-CM5, (**l**) GFDL-CM3, (**m**) GFDL-ESM2G, (**n**) GFDL-ESM2M, (**o**) inmcm4, (**p**) IPSL-CM5B, and (**q**) ENSMEAN-MOD for the historical.

The intra- and interannual Hovmöller diagrams of the EIR depicted in Figure 7 illustrate the variation of malaria over the years as a function of the months. The results of the reference data show the maximum of the EIR, which is observed in September. High malaria transmission is clearly evident in August–October (Figure 7a–e).

Some models, such as CMCC-CMS, GFDL-ESM2G, and IPSL-CM5B, show an increasing evolution of malaria over the years (see Figure 7j,m,p). Other models, such as CanESM2, CMCC-CM, and inmcm4, show a decrease in malaria over time (see Figure 7g,i,o). The CSIRO, CNRM-CM5, GFDL-CM3, and GFDL-ESM2M models show alternating high and low years of malaria transmission (see Figure 7h,k,L,n).The simulations of the CMIP5 GMCs show a much larger signal than the signal obtained with the reference data. In other words, the simulations overestimate the number of infectious mosquito bites per man per month over time. The period of strong transmission (between August, September, and October) is reproduced by the models, as is the maximum peak (obtained in September). The ACCESS1–3 model and the ensemble mean of all CMIP5 models (ENSMEAN-MOD) appear to provide a fairly good representation of the variation in malaria over time (Figure 7f,q).

For the variation of malaria transmission from one year to another according to latitudes, we note that the periods 1986–1989, 1992–2000, and 2003–2005 are marked by a strong transmission of malaria that extends from latitudes 12° N to 15° N. As obtained with the simulations of the reference data, the results of the simulations of the CMIP5 models showed a strong transmission of malaria extending from latitudes 12° N to 15° N during the periods 1986–1989, 1992–2000, and 2003–2005. (See Figure S2 in the Supplementary Material).

**Figure 8:**
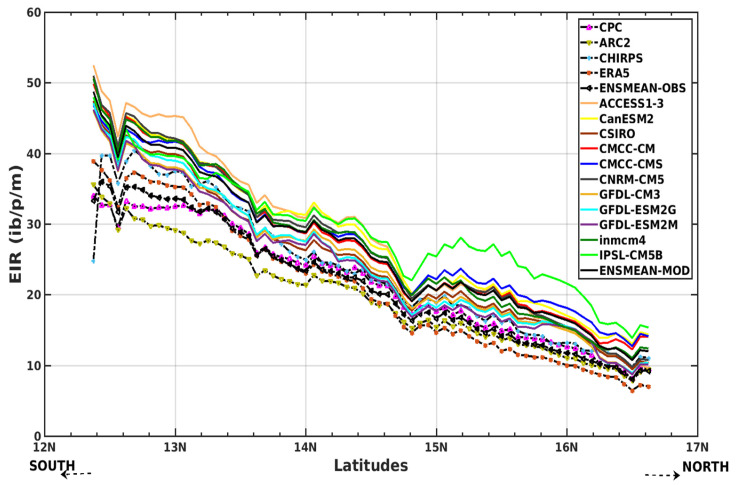
Mean EIR (in ib/p/m, i.e., infectious bites per person per month) meridian gradient of malaria in Senegal for the period 1983–2005: Simulations of the VECTRI model forced by rainfall and temperature of CPC, ARC2, CHIRPS, ERA5, ENSMEAN-OBS (the reference data used as observation data) for evaluation and bias-corrected CMIP5 GCM models: from ACCESS1–3, CanESM2, CSIRO, CMCC-CM, CMCC-CMS, CNRM-CM5, GFDL-CM3, GFDL-ESM2G, GFDL-ESM2M, inmcm4, IPSL-CM5B, ENSMEAN-GCM for historical.

Figure 8 shows the variation of the EIR as a function of latitudes for the four reference datasets and their overall mean (CPC, ARC2, CHIRPS, ERA5, and ENSMEAN-OBS). We see a decrease in EIR from the southern latitudes to the northern latitudes of Senegal. Additionally, the CHIRPS data gives the highest EIR values from 12° N to 17° N. As for the others, the ARC2 data provide the smallest EIR values from 12° N down to 14.75° N; beyond this value, the ERA5 data presents the lowest values.

The results of the CMIP5 models show a very clear meridian variation of malaria. The models clearly reproduced the south–north gradient. The simulations show a clearer differentiation of the EIR further south than north for all models. The IPSL-CM5B model deviates from other models at latitudes 14.75 N up to 17 N.

**Figure 9:**
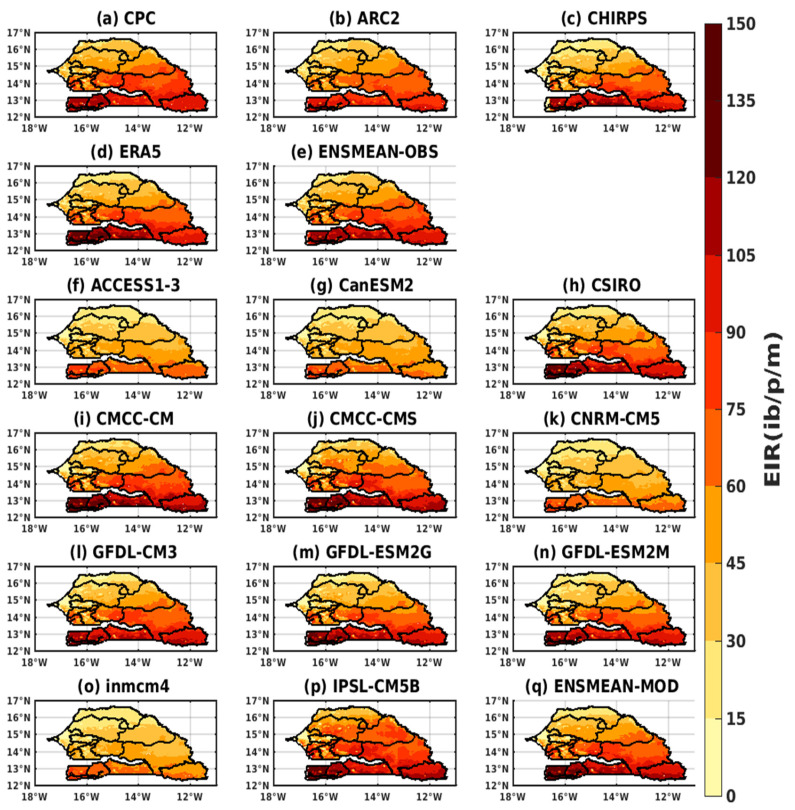
Spatial distribution of the EIR (in ib/p/m i.e., infectious bites per person per month) of malaria in September, October, and November in Senegal for the period 1983–2005: Simulations of the VECTRI model forced by rainfall and temperature of the assessment data (**a**) CPC, (**b**) ARC2, (**c**) CHIRPS, (**d**) ERA5, and (**e**) ENSMEAN-OBS (the reference data used as observation) for validation and bias-corrected CMIP5 GCM models: (**f**) ACCESS1–3, (**g**) CanESM2, (**h**) CSIRO, (**i**) CMCC-CM, (**j**) CMCC-CMS, (**k**) CNRM-CM5, (**l**) GFDL-CM3, (**m**) GFDL-ESM2G, (**n**) GFDL-ESM2M, (**o**) inmcm4, (**p**) IPSL-CM5B, and (**q**) ENSMEAN-MOD for historical.

The spatial distribution of RIA (Figure 9) shows a clear difference in signal intensity between the northern and southern regions of Senegal. We find, with the reference data (Figure 9a–e), that the regions located in the southwestern and southeastern parts are more affected by malaria compared to the other regions for the different data as well as the global average (ERA5, CPC, ARC2, CHIRPS, and ENSMEAN-OBS). As obtained with precipitation, the transmission extends from south to north up to latitudes 15° N.

We find that the simulations of the CMIP5 models show more of the difference between the south and the north (Figure 9f–q). Malaria transmission is much higher in the south than in the north. The CMCC-CM, CMCC-CMS, and IPSL-CM5B models show the presence of malaria over almost all of the territory except for a small part of the north, i.e., latitudes above 16 N (Figure 9i,j,p) unlike what was found with the validation data (Figure 9f–q). Concerning the GFDL-CM3, GFDL-ESM2G, GFDL-ESM2M, inmcm4, and ENSMEAN-MOD models (Figure 9h,l–n,q), we note that their results are close to those of the validations. As for the ACCESS1–3, CanESM2, CNRM-CM5, and inmcm4 models (Figure 9f,g,k,o), the distribution is weak on the territory, but the unequal distribution between the south and the north has been found.

#### 3.2.2. Taylor Diagram for Rainfall, Temperature and EIR Variable

**Figure 10:**
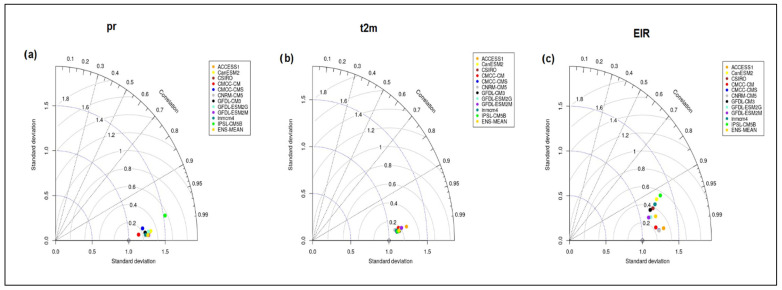
Taylor plots displaying rainfall statistics, temperature, and simulated EIR, comparing monthly bias-corrected CMIP5 data to baseline monthly climate data: (**a**) precipitation (pr), (**b**) temperature (t2m), (**c**) Simulated EIR.

Statistical performance measures are summarized in the Taylor diagram in Figure 10. The root-mean-square difference (RMSE), the pattern correlation I(r), and the standard deviation (STD) are three statistical measures that are used with the Taylor diagram, computed between bias-corrected CMIP5 results and the ensemble mean (ENSMEAN-OBS) of the four types of rainfall (ERA5, CPC, ARC2, and CHIRPS) (Figure 10a) and ERA5 temperature (Figure 10b), which are used as a point of reference. Figure 10c shows the Taylor diagram for simulated EIR using bias-corrected CMIP5 and simulated EIR using the reference climate data. The Pearson correlation coefficient is indicated by the grey dashed lines, the root mean square error (RMSE) by the grey outlines, and the standard deviation (STD) by the blue outlines. The models to be compared are shown by colored points and the observation by the uncolored point on the x-axis. The CMCC-CM provides a very faithful representation of the monthly mean rainfall of the individual bias-corrected CMIP5 models and their ensemble mean compared with the reference data. The inmcm4, CNRM-CM5, and CMCC-CMS models provide a good representation of the temporal variation of the annual rainfall cycle. Alone, the IPSL-CM5B model has a large positive bias compared to the observed data. ACCES1, GFDL-ESM2G, GFDL-ESM2M, and CanESM2 perform relatively well against reference data with low root mean square errors and high correlations. The ensemble mean (ENSMEAN-OBS) also provides good results but always overestimates the basic rainfall.

The quantitative diagnostics in Figure 10b show that the models are very strongly correlated (r > 0.98) with the reference data for the monthly mean temperatures and have a normalized standard deviation close to 1 (slightly greater than 1). Most models (CMCC-CM, CMCC-CMS, CNRM-CM5, ACCESS1, GFDL-ESM2M, GFDL-ESM2G, and GFDL-CM3) represent the annual temperature cycle well. The ACCESS1–3 model shows the largest bias against the observed data. However, it is important to note that CMIP5 models overestimate the annual temperature cycle (Figure 10b).

Figure 10c provides a summary of the relative skill of the 11 models in simulating malaria transmission through the EIR variable. The results show a strong correlation for all 11 models (values greater than 0.9). Despite this correlation, we note the presence of a strong dispersion among the models but also more or less remarkable errors for the IPSL-CM55B, CanESM2, and inmcm4 models. The CMCC-CM, CMCC-CMS, CNRM-CM5, and ACCESS1–3 models show strong correlations (0.99). In addition to the correlation, the CMCC-CM, CMCC-CMS, and CNRM-CM5 models present the lowest root mean square error (RMSE) values (0.25). The GFDL-ESM2M, GFDL-ESM2G, and GFDL-CM3 models are less dispersed (lower standard deviation values) with strong correlation values (greater than 0.95). The GFDL-CM3 model, compared to the two other versions of the model, simulates the EIR variable less well, with high RMSE values, a weaker correlation, and a significant standard deviation. Most models overestimate the magnitude of the entomological inoculation rate. The results show that the CMCC-CM and CNRM-CM5 models perform better at representing the EIR variable. It should also be noted that the CMCC-CMS models present scores very close to those of the CMCC-CM.

In summary, in Figure 10a,b, we note that for all models, r is greater than 0.99, RMSE is lower than 0.2, and the STD is approximately around 1.25. These three statistical values illustrate the performance of individual bias-corrected CMIP5 in reproducing precipitation observations (ERA5) and the ad value by using these data as input for VECTRI simulations. For the simulated EIR (Figure 10c), there are more discrepancies between the models, but the r values are also all greater than 0.95, the RMSE values comprise between 0.1 and 0.6, and the STD is also between 1 and 1.5.

### 3.3. Projected Changes in Malaria Index (EIR)

**Figure 11:**
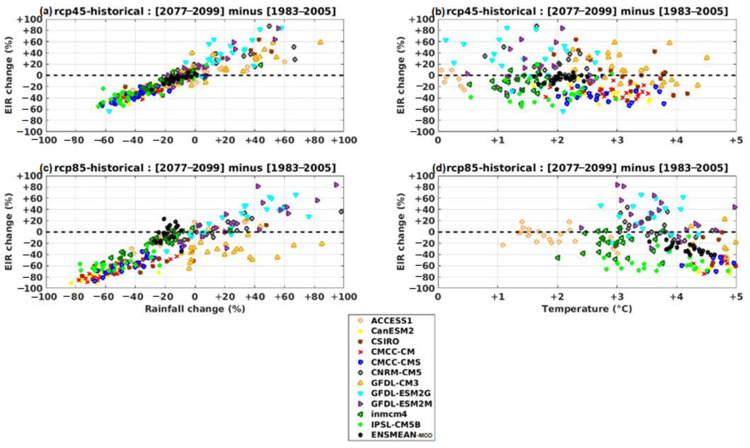
Relative changes in VECTRI-simulated malaria transmission versus percent precipitation change (left) and absolute mean surface temperature change (right) for RCP45 and RCP85 scenarios from CMIP5 GCM data corrected for the far future: CanESM2, CSIRO, CMCC-CM, CMCC-CMS, CNRM-CM5, GFDL-CM3, GFDL-ESM2G, GFDL-ESM2M, inmcm4, IPSL-CM5B, ENSMEAN-MOD.

The results in Figure 11 show the effect of precipitation and temperature changes on malaria transmission for the far future period. We calculated, over this period of 23 years, for each model the future-historical differences of EIR in percentage, as well as the future-historical differences of temperatures (in absolute) and the future-historical differences of rain (in%). We then traced the relationship between the climatic variables (precipitation and temperature) and the malaria variable (EIR).

We found that malaria increases linearly with increasing rainfall in the future for both scenarios (rcp45 and rcp85) for the ensemble of models used and their ensemble mean (Figure 11a,c). In other words, in general, if total seasonal rainfall increases, malaria transmission will increase and if rainfall decreases, transmission will also decrease. However, heavy rains can flush the female mosquitoes’ eggs deposited on the water surfaces. On the other hand, intermittent rainfall (or showers) can enhance the development of the mosquito vector population. The increase in temperature has a negative impact, especially in hot areas where infection currently occurs with temperatures favorable to transmission (Figure 11b,d). On the other hand, a warming climate may increase the incidence of malaria in colder mountainous areas. The more the temperature increases, the more the transmission tends to decrease. This negative drop is more visible in the rcp85 scenario. Most models show the impact of climate change on malaria transmission.

The same result was obtained in the near and medium future (see Figures S3 and S4 in the Supplementary Material).

**Figure 12:**
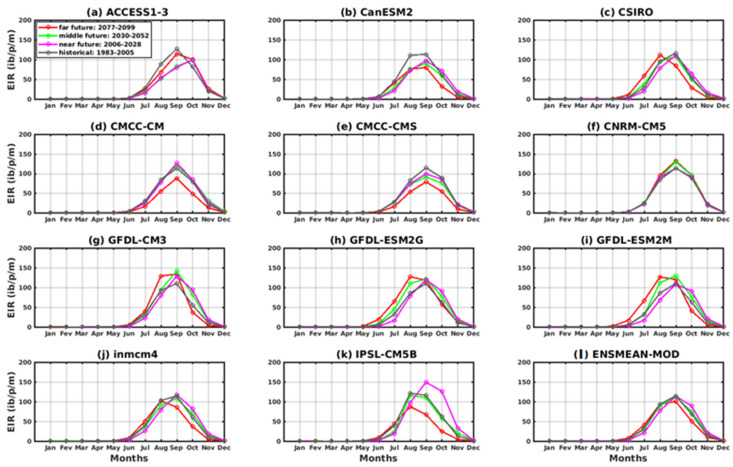
The annual EIR cycle of malaria in Senegal for the period 2006–2100 (historical: 1976–2005 grey curve, near future: 2006–2035 pink curve, middle future: 2036–2065 green curve, far future: 2066–2095 red curve. Simulations of the VECTRI model forced by rainfall and temperature CMIP5 GCM models corrected: (**a**) ACCESS1–3, (**b**) CanESM2, (**c**) CSIRO, (**d**) CMCC-CM, (**e**) CMCC-CMS, (**f**) CNRM-CM5, (**g**) GFDL-CM3, (**h**) GFDL-ESM2G, (**i**) GFDL-ESM2M, (**j**) inmcm4, (**k**) IPSL-CM5B, and (**l**) ENSMEAN-MOD for RCP45.

**Figure 13:**
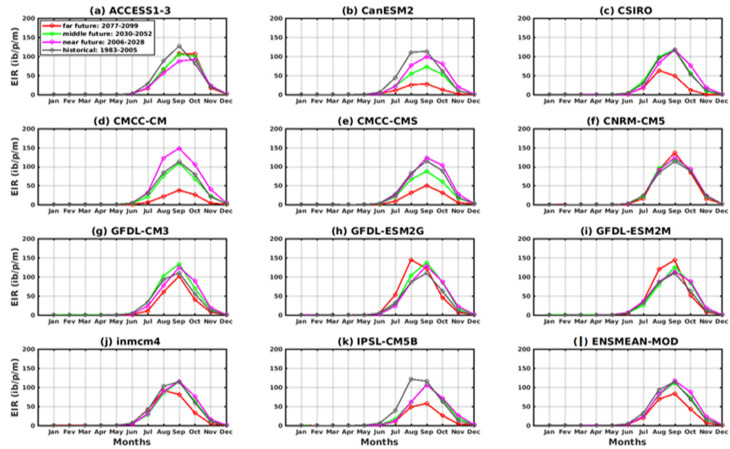
The annual EIR cycle of malaria in Senegal for the period 2006–2100 (historical: 1976–2005 grey curve, near future: 2006–2035 pink curve, middle future: 2036–2065 green curve, far future: 2066–2095 red curve). Simulations of the VECTRI model forced by rainfall and temperature CMIP5 GCM models corrected: (**a**) ACCESS1–3, (**b**) CanESM2, (**c**) CSIRO, (**d**) CMCC-CM, (**e**) CMCC-CMS, (**f**) CNRM-CM5, (**g**) GFDL-CM3, (h) GFDL-ESM2G, (**i**) GFDL-ESM2M, (**j**) inmcm4, (**k**) IPSL-CM5B, and (**l**) ENSMEAN-MOD for RCP85.

All models reproduced the annual cycle well in these scenarios. The peak is obtained in September over the historical period (1983–2005) with the two scenarios for all models. For the period of the near future (2006–2035), most of the models show a peak in September except the ACCESS1–3 model (Figure 12a and Figure 13a), which exhibits in this period a peak in October under the two scenarios used. In the period of the middle future (2036–2065) as well as the far future (2066–2095), the ACCESS1–3 model gives the maximum of the EIR in October, and other models in August such as CSIRO, GFDL -ESM2G, GFDL-ESM2M, inmcm4, IPSL-CM5B (Figure 12c,h–k), and a maximum in September prevailed for the rest of the models, including CanESM2, CMCC-CM, CMCC-CMS, CNRM-CM5, and GFDL-CM3 (Figure 12b,d–g). However, there are discrepancies in the peak amplitude between the models. In Figure 13, in general, there is agreement in the results for the RCP4.5 and RCP8.5 scenarios for the historical, near future, and middle future periods regarding the annual profile despite discrepancies in the range. For the far future, only the CSIRO and GFDL-ESM2G models (Figure 13c,h) agree on the period of the far future.

The representation of the variation of malaria transmission during the year according to the latitudes of the simulations shows that the hotspots in the malaria epidemics are located in the southern regions (up to 15° N) for the CanESM2 models, CSIRO, GFDL-ESM2M, and IPSL-CM5B for the RCP4.5 scenario. Similar observations were observed with the RCP8.5 scenario for the CSIRO and GFDL-ESM2M models. The period of high malaria transmission is observed from August to October for all models of the RCP4.5 scenario. Some models, including ACCESS1–3, CNRM-CM5, and inmcm4, show the period of high transmission outbreaks from July to November for the RCP8.5 scenario (see Figures S5 and S6 in the Supplementary Material).

**Figure 14:**
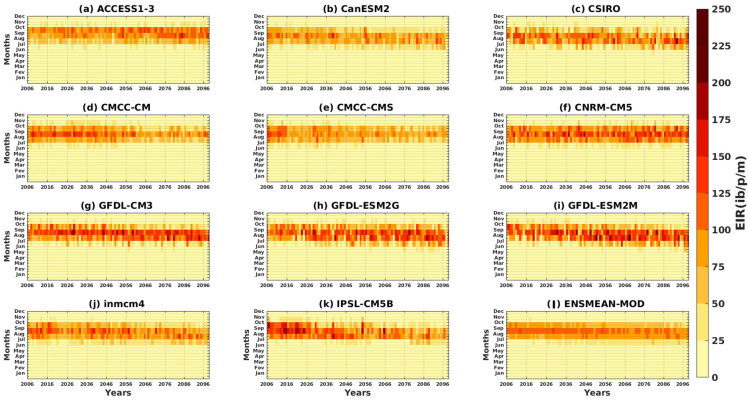
Hovmöller diagram intra/inter of the annual cycle of EIR in Senegal for the period 2006–2100. Simulations of the VECTRI model forced by rainfall and temperature of bias-corrected CMIP5 GCM models: (**a**) ACCESS1–3, (**b**) CanESM2, (**c**) CSIRO, (**d**) CMCC-CM, (**e**) CMCC-CMS, (**f**) CNRM-CM5, (**g**) GFDL-CM3, (**h**) GFDL-ESM2G, (**i**) GFDL-ESM2M, (**j**) inmcm4, (**k**) IPSL-CM5B, and (**l**) ENSMEAN-MOD for RCP45.

**Figure 15:**
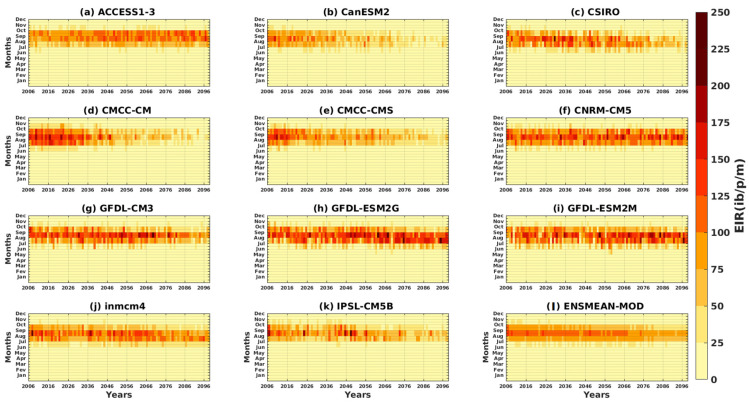
Hovmöller diagram intra/inter of the annual cycle EIR in Senegal for the period 1983–2005. Simulations of the VECTRI model forced by rainfall and temperature bias-corrected CMIP5 GCM models: (**a**) ACCESS1–3, (**b**) CanESM2, (**c**) CSIRO, (**d**) CMCC-CM, (**e**) CMCC-CMS, (**f**) CNRM-CM5, (**g**) GFDL-CM3, (**h**) GFDL-ESM2G, (**i**) GFDL-ESM2M, (**j**) inmcm4, (**k**) IPSL-CM5B, and (**l**) ENSMEAN-MOD for RCP85.

The intra- and interannual variations of the entomological inoculation rate (EIR) described in Figure 14 and Figure 15 illustrate the number of infectious mosquito bites per man and per month over the years. The models agree on a maximum EIR in September, whatever the scenario, except for ACCESS1–3 (Figure 14a and Figure 15a). An increase in malaria transmission in the far future (2066–2095) compared to the near future (2036–2065) is found in the simulations ACCESS1–3, CSIRO, CNRM-CM5, GFDL-CM3, GFDL-ESM2G, and GFDL-ESM2M (Figure 14a,c,f–i), while the other models, such as CanESM2, CMCC-CM, CMCC-CMS, inmcm4, and IPSL-CM5B, predict a decrease for the RCP4.5 scenario (Figure 14d,e,j,k and Figure 15d). The decrease in malaria in the future with the models cited above is much more visible under the RCP8.5 scenario (Figure 15b,d,e,j,k).

The variation in malaria transmission from one year to the next as a function of latitude shows the decrease in malaria in the territory of Senegal for the different CMIP5 models in the two scenarios (RCP4.5 and RCP8.5), but also in the years to come (2066–2100). This decrease is more significant with the RCP8.5 scenario (see Figures S7 and S8 in the Supplementary Material).

**Figure 16:**
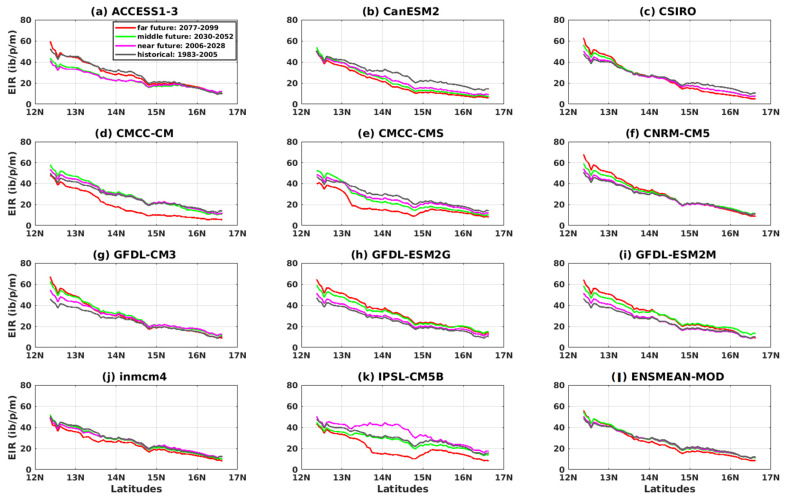
Mean EIR meridian gradient of malaria in Senegal for the period 2006–2100 (historical: 1983–2005 grey curve, near future: 2006–2028 pink curve, middle future: 2030–2052 green curve, far future: 2077–2099 red curve). Simulations of the VECTRI model forced by rainfall and temperature of bias-corrected CMIP5 GCM models: (**a**) ACCESS1–3, (**b**) CanESM2, (**c**) CSIRO, (**d**) CMCC-CM, (**e**) CMCC-CMS, (**f**) CNRM-CM5, (**g**) GFDL-CM3, (**h**) GFDL-ESM2G, (**i**) GFDL-ESM2M, (**j**) inmcm4, (**k**) IPSL-CM5B, and (**l**) ENSMEAN-MOD for RCP45.

**Figure 17:**
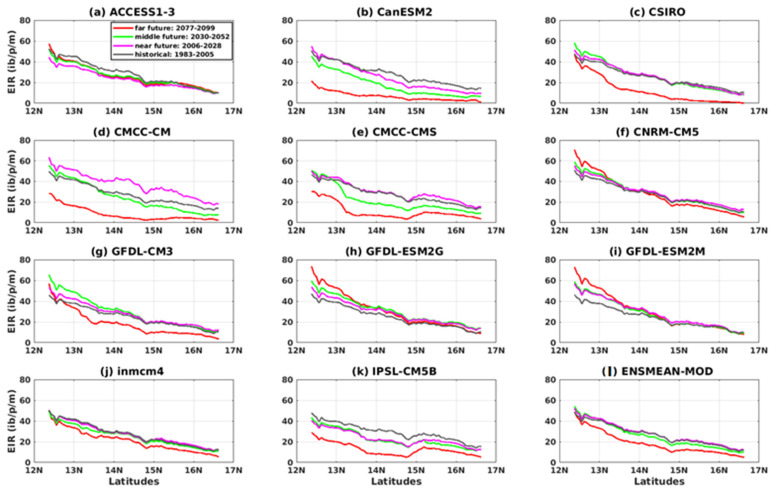
Mean EIR meridian gradient of malaria in Senegal for the period 2006–2100 (historical: 1983–2005 grey curve, near future: 2006–2028 pink curve, middle future: 2030–2052 green curve, far future: 2077–2099 red curve): Simulations of the VECTRI model forced by rainfall and temperature of bias-corrected CMIP5 GCM models (**a**) ACCESS1–3, (**b**) CanESM2, (**c**) CSIRO, (**d**) CMCC-CM, (**e**) CMCC-CMS, (**f**) CNRM-CM5, (**g**) GFDL-CM3, (**h**) GFDL-ESM2G, (**i**) GFDL-ESM2M, (**j**) inmcm4, (**k**) IPSL-CM5B, and (**l**) ENSMEAN-MOD for RCP85.

The results show a very clear meridian variation of malaria for the future, with a south–north gradient (Figure 16 and Figure 17). The simulations show a clearer differentiation of the EIR for the different periods, more in the south than in the north. The CanESM1, CSIRO, and CMCC-CM models (Figure 17b–d) predict an entomological inoculation rate that would be relatively low in the north in the future for the RCP8.5 scenario. However, the difference in periods with the RCP8.5 scenario (Figure 17) is much clearer than that found with the RCP4.5 scenario (Figure 16).

## 4. Discussion

The VECTRI simulations based on bias-corrected CMIP5 show repercussions on malaria transmission in Senegal. The simulations showed that climate influences the epidemiology and geographic distribution of malaria. Studies have shown that malaria transmission follows rainfall patterns [53,63,64,65,66]. High malaria transmission is found during the rainy season. The EIR peak prevailed in September. The rainfall peak in August is generally followed by the malaria peak in about one to two months [12,67,68]. This is explained by the fact that the water bodies develop where the mosquitoes lay. The larval development in aquatic environments can last according to the temperature: 1 to 3 weeks in tropical areas and several weeks or months in temperate areas [7,69]. In addition, the infected person takes time before developing symptoms [70]. In Senegal, like rainfall, malaria transmission follows the decreasing variation in the south–north direction. The southern part of Senegal (humid and rainy) is the area most affected by malaria compared to the northern part of the country (dry and arid). The aridity limits the survival and ability of adult *Anopheles* vectors to contribute to parasite transmission [71]. The unequal distribution of rain in the Senegalese territory is due to the weather that the monsoon takes to reach the country [72,73]. The trade winds from a cyclone in Sainte Hélène, which creates the monsoon loaded with humidity, reach the country in the month of April. This meteorological phenomenon crosses the territory in the northeast direction, thus implying the start of the rainy season in Senegal. For the southern regions, the season starts in May, and for the central and northern regions, it starts around June or July. The maximum rainfall in Senegal is generally obtained in August [29,74]. In addition to rain, humidity, and hot periods, high vegetation coverage, and optimal malaria transmission temperatures (25 °C, 6 °C lower than previous models) [75] also promote transmission. Some studies argue that temperature fluctuations significantly affect both the life expectancy or completion of the mosquito life cycle and the development of sporogonic stages in the body of the malaria patient [53,76,77]. At warmer temperatures, mosquitoes increase the rate of parasite transmission, reach sexual maturity earlier, and obtain more human blood [78]. In Senegal, the main species found are *Plasmodium falciparum* and a few rare cases of *Plasmodium ovale* and *Plasmodium malariae*. Among the different species that infect humans, *Plasmodium falciparum* (~98%) is the one that causes the most severe cases in Senegal. A movement of identical parasites from one region to another and from one year to another is also noted in the country [6,7].

The period 1983–2005 (historical), which is the reference period, was characterized by an alternation between wet years and dry ones with a continuity of strong rainfall instability. However, that did not prevent years of high, medium, and low malaria transmission. Malaria varies depending on the years of extreme weather events such as floods and heat waves [79].

Temperatures have a greater impact than rainfall on malaria transmission because of interannual variability [3]. The variation in EIR is attributed to the decrease in rainfall since the 1970s for some studies [67]; for others, this variation is due to the variability of temperatures [80].

However, the results predicted by the models on the occurrence of malaria by 2100 with the RCP8.5 scenario considered a high emission scenario and the RCP4.5 scenario considered an intermediate mitigation scenario have differences. Some models (ACCESS1–3, CSIRO, CNRM-CM5, GFDL-CM3, GFDL-ESM2G, and GFDL-ESM2M) predict the increase in malaria regardless of the scenario (RCP4.5 and RCP8.5) (Figure 14a,c,f–i and Figure 15a,c,f–i). On the other hand, other models (CanESM2, CMCC-CM, CMCC-CMS, inmcm4, and IPSL-CM5B) predict a decrease for the RCP4.5 scenario (Figure 14b,d,e,j,k). The decrease in malaria projected in the future with these models is much more visible under the RCP8.5 scenario (Figure 15b,d,e,j,k).

This reduction in malaria with these climate models could be explained by the fact that they actually show a downward trend in rainfall [81] one of the aspects that can, on the one hand, justify the reduction in malaria in the far future, in particular with the extreme scenario RCP8.5. The other aspect is that the optimal temperature of transmission in Africa is 25 °C, and beyond 28 °C, it declines [82]. We would expect temperature increases [83] that will be larger than the optimum in the far future, especially with the RCP8.5. Hot temperatures could negatively impact mosquito survival, and heavy flooding could cause larvae to be displaced [84].

The negative impact of climate change on malaria transmission is a very robust finding, especially for long-term projections (2077–2099). About 70% of IPCC climate scenarios have shown that warming is expected to exceed +2 °C for sub-Saharan West Africa. In addition, a reduction in precipitation is expected in the far future [85].

Notwithstanding, an increase in malaria transmission due to climate change should not be expected in areas where transmission is stable or unstable at low levels [86].

In contrast, future climate warming could increase malaria incidence in colder mountainous regions while decreasing incidence in already warm regions with average temperatures above 25 °C [87], as the results of RCP4.5 show. These observations agree with the conclusions according to which the epidemic fringe would have shifted towards the south for most models of malaria. The climate is expected to become unfavorable in the northern Sahel in the far future [15]. So, the drop in signal in the far future is not a surprising result, as research has shown that malaria transmission may gradually decrease in West Africa in the far future [88]. Such a decrease in malaria seems to be associated with climate change and the consideration of socio-economic factors [89,90]. A decrease in the simulated conditions of malaria in the Sahel has been shown [90] whatever the period and the scenario considered, which are linked to a temperature effect.

In the case of years of high rainfall, there may be periods in which transmission continues until the beginning of the dry season. It is ensured by the reproduction of the *Anopheles* vector, which continues to improve thanks to the strong heat of October, the presence of breeding sites, and plant cover. This allows the eggs to resist drought by remaining latent in the moist soil [91], hence the presence of malaria outside the rainy season (October) shown by some models. Sometimes the late start of the season contributes to the displacement of the maximum EIR in October. However, heavy flooding could cause the displacement or destruction of the larvae, or, in other words, the reduction of the transmission.

The results obtained using projections given by different GCM models are of paramount importance. However, according to our results, malaria will be more important in the RCP4.5 scenario, which is less restrictive than the RCP8.5 scenario.

Relating climate change to malaria projections is not a simple matter. There are many uncertainties in the projection of malaria-related temperatures and rainfall. The uncertainties come from the imperfection of climate models, the bias correction methods, the choice of climate parameters, the choice of an observation-based reference dataset on bias correction, climate change scenarios, and the malaria model (we used VECTRI).

The present study focuses on three sources (climate models, bias correction methods, and malaria models).

In the chosen GCMs, depending only on simulation availability, we have not evaluated their projection skills in the Sahel area and were not based on their spatial resolution. Due to the coarse spatial resolution of GCMs, we certainly neglected some atmospheric processes that can affect temperature and rainfall parameters.

Using CFD-t bias correction, we applied this method independently for each of the two variables. However, doing that may cause the spatial coherency and dependence among variables to be removed by the application of univariate calibrations. Other recent studies to address this challenge are focused on multivariate correction and spatial and/or temporal dependences [92,93]. Other more sophisticated methods using multivariate correction were also recently developed.

Finally, we used the new malaria model VECTRI, which was run and evaluated in the Sahel domain.

Nevertheless, our paper shows that it is crucial to use multi-climate models to evaluate the response of malaria to climate change, which has been overlooked by previous malaria studies in West Africa.

## 5. Conclusions

In this study, the VECTRI malaria model is used to simulate the spatio-temporal variation of the malaria parameter (EIR) in Senegal. The simulations are based on ERA5, CPC, ARC2, and CHIRPS data to assess and CMIP5 global climate models, separating historical data and projection data. The two scenarios (RCP8.5 and RCP4.5) made it possible to know the presence and evolution of malaria in the future. This means that the model can reproduce the effect of climate change on malaria transmission. The study revealed that the maximum of the EIR (obtained in September) follows the rainfall regimes (obtained in August), with a strong period of transmission generally located between August and October for a major part of the models and July and November for certain models. Precipitation provides breeding grounds for mosquitoes, while temperature impacts larval and mosquito life cycles. Even if our results reveal a decrease in malaria transmission in Senegal in the far future, the burden of malaria will likely increase in many parts of the world due to climate change. The decrease in rainfall in the two scenarios (RCP8.5 and RCP4.5) associated with very hot temperatures might imply a reduction in the mosquito population. However, these results must be interpreted with caution, as there are still uncertainties related to both the disease model and the GCM projections for the future. Given the wide divergence between models, it is usually advisable not to base assessments of future climate change on the results of a single model used in isolation; so, we can conclude by suggesting working on an average of a set of different GCM models (multi-model ensemble mean) in addition to individual models if a comparison is needed.

These results will assist in decision-making and allow for the establishment of preventive surveillance systems for local climate-sensitive diseases including malaria, especially in areas currently free of malaria and expected to be affected by the disease in the future. Future research will be done based on the combination of RCPs with the shared socio-economic pathway (SSP), which considers key scenario factors such as socio-economic growth, urbanization, and population for the estimation of future malaria.

## Figures and Tables

**Table 1:** Datasets: this table summarizes the available datasets.

Climate Datasets	Definition	Resolution	References
ERA5 (Rainfall at temperature)	European ReAnalysis	0.25° × 0.25°	[30]
CHIRPS (Rainfall)	Climate Hazards InfraRed Rainfall with Station data	0.05° × 0.05°	[32]
ARC2 (Rainfall)	Africa Rainfall Climatology, version 2	0.1° × 0.1°	[33]
CPC (Rainfall)	Climate Prediction Center	0.25° × 0.25°	[34]

**Table 2:** List of the 11 global circulation models (GCMs) used in the study.

Model	Institute	Resolution	Reference
ACCESS1–3	Australian Community Climate and Earth System Simulator, Australia	1.25° × 1.9°, L38	[43]
CanESM2	Canadian Centre for Climate Modeling and Analysis, Canada	2.8° 9 × 2.8°, L35	[44]
CMCC-CM	Centro Euro-Mediterraneo per I Cambiamenti Climatici, Italy	0.75° × 0.75°, L31	[45]
CMCC-CMS	Centro Euro-Mediterraneo per I Cambiamenti Climatici, Italy	0.75° × 0.75°, L31	[45]
CNRM-CM5	Centre National de Recherches Météorologiques, France	1.4° × 1.4°, L31	[46]
CSIRO-Mk3–6-0	CSIRO-QCCCE, Australia	1.9° × 1.9°, L18	[47]
GFDL-CM3	Geophysical Fluid Dynamics Laboratory-Climate Model version 3, USA	2° × 2.5°, L48	[48]
GFDL-ESM2G	Geophysical Fluid Dynamics Laboratory-Earth System Models version 2G, USA	2° × 2.5°, L48	[49]
GFDL-ESM2M	Geophysical Fluid Dynamics Laboratory Earth System Models version 2M, USA	2° × 2.5°, L48	[50]
Inmcm4	Institute for Numerical Mathematics, Russia	2° × 1.5°, L21	[51]
IPSL-CM5B-LR	Institut Pierre-Simon Laplace, France	1.895° × 3.75°, L39	[52]

## Data Availability

The reanalysis data used in this work was obtained from the Climate Data Store (CDS) of the European Center for Medium-Range Weather Forecasts (ECMWF), i.e., this link: https://cds.climate.copernicus.eu/cdsapp#!/dataset/reanalysis-era5-single-levels?tab=form. The data is downloaded using the CDS API (Python script). CHIRPS, ARC2, and CPC data were downloaded programmatically into a bash file.

## Supplementary Materials

The following supporting information can be downloaded at: https://www.mdpi.com/article/10.3390/tropicalmed8060310/s1. Figure S1: Hovmöller diagram of the EIR of malaria in Senegal for the period 1983–2005: Simulations of the VECTRI model forced by rainfall and temperature of the assessment data; Figure S2: Hovmöller diagram interannual of the EIR of malaria in Senegal for the period 1983–2005: Simulations of the VECTRI model forced by rainfall and temperature of the assessment data; Figure S3: Relative changes in VECTRI-simulated malaria transmission versus percent precipitation change (left) and absolute mean surface temperature change (right) for RCP45 and RCP85 scenarios from bias-corrected CMIP5 GCM models for the near future: CanESM2, CSIRO, CMCC-CM, CMCC-CMS, CNRM-CM5, GFDL-CM3, GFDL-ESM2G, GFDL-ESM2M, inmcm4, IPSL-CM5B, ENSMEAN-MOD; Figure S3: Relative changes in VECTRI-simulated malaria transmission versus percent precipitation change (left) and absolute mean surface temperature change (right) for RCP45 and RCP85 scenarios from bias-corrected CMIP5 GCM models for the near future: CanESM2, CSIRO, CMCC-CM, CMCC-CMS, CNRM-CM5, GFDL-CM3, GFDL-ESM2G, GFDL-ESM2M, inmcm4, IPSL-CM5B, ENSMEAN-MOD; Figure S5: Hovmöller diagram of the annual EIR cycle of malaria in Senegal for the period 2006–2100: Simulations of the VECTRI model forced by rainfall and temperature bias-corrected CMIP5 GCM models; Figure S6: Hovmöller diagram of the annual EIR cycle of malaria in Senegal for the period 2006–2100: Simulations of the VECTRI model forced by rainfall and temperature bias-corrected CMIP5 GCM models; Figure S7: Hovmöller diagram of the interannual EIR cycle of malaria in Senegal for the period 2006–2100: Simulations of the VECTRI model forced by rainfall and temperature bias-corrected CMIP5 GCM models; Figure S8: Hovmöller diagram of the interannual EIR cycle of malaria in Senegal for the period 1983–2005: Simulations of the VECTRI model forced by rainfall and temperature bias-corrected CMIP5 GCM models.
