# Supplementary material for: Bias-Corrected CMIP5 Projections for Climate Change and Assessments of Impact on Malaria in Senegal under the VECTRI Model
Source: Trop Med Infect Dis. 2023 Jun 6;8(6):310. doi: 10.3390/tropicalmed8060310 (PMC10300711; doi:10.3390/tropicalmed8060310)
Supplement: Supplementary file 1 [file tropicalmed-08-00310-s001.zip › tropicalmed-2065461-supplementary.pdf]

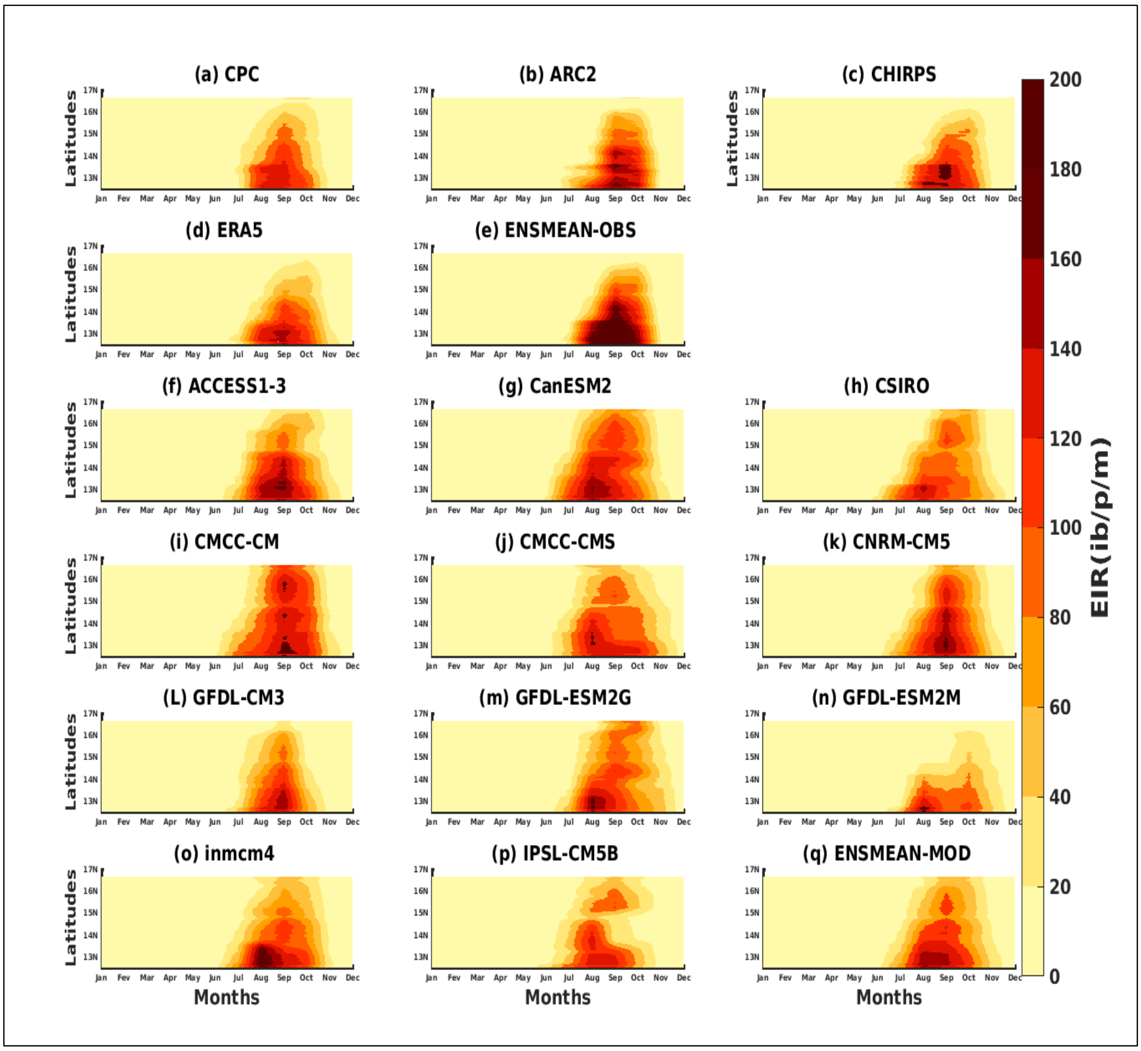

Figure S1: Hovmöller diagram of the EIR of malaria in Senegal for the period 1983-2005: Simulations of the VECTRI model forced by rainfall and temperature of the assessment data: a) CPC, b) ARC2, c) CHIRPS, d) ERA5, and e) ENSMEAN-OBS (the reference data used as observation data) for validation and bias-corrected CMIP5 GCM models: a) ACCESS1-3, b) CanESM2, c) CSIRO, d) CMCC-CM, e) CMCC-CMS, f) CNRM-CM5, g) GFDL-CM3, h) GFDL-ESM2G, i) GFDL-ESM2M, j) inmcm4, k) IPSL-CM5B, l) ENSMEAN-MOD for historical

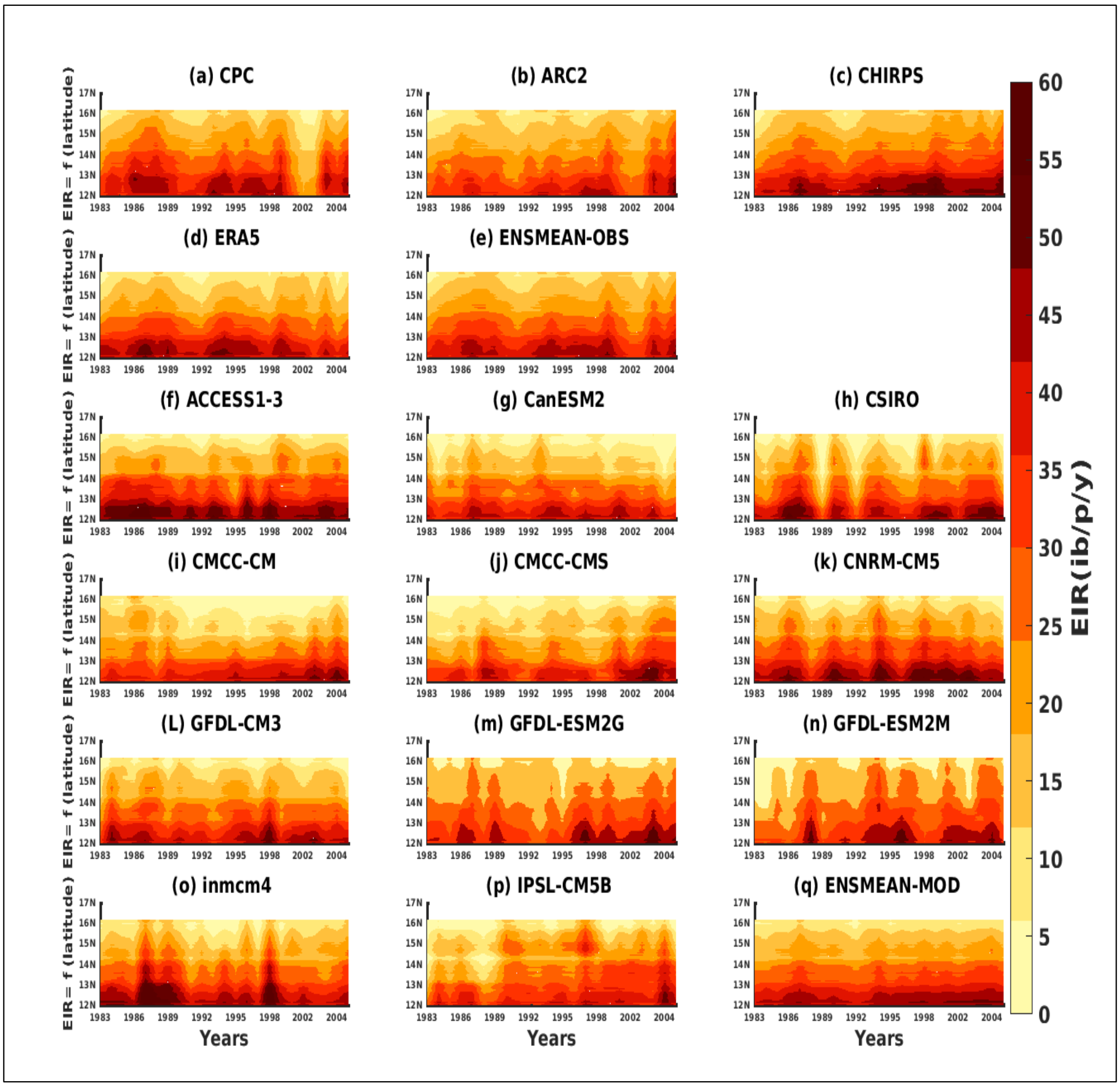

Figure S2: Hovmöller diagram interannual of the EIR of malaria in Senegal for the period 1983-2005: Simulations of the VECTRI model forced by rainfall and temperature of the assessment data: a) CPC, b) ARC2, c) CHIRPS, d) ERA5, and e) ENSMEAN-OBS (the reference data used as observation data) for validation and bias-corrected CMIP5 GCM models: a) ACCESS1-3, b) CanESM2, c) CSIRO, d) CMCC-CM, e) CMCC-CMS, f) CNRM-CM5, g) GFDL-CM3, h) GFDL-ESM2G, i) GFDL-ESM2M, j) inmcm4, k) IPSL-CM5B, l) ENSMEAN-MOD for historical

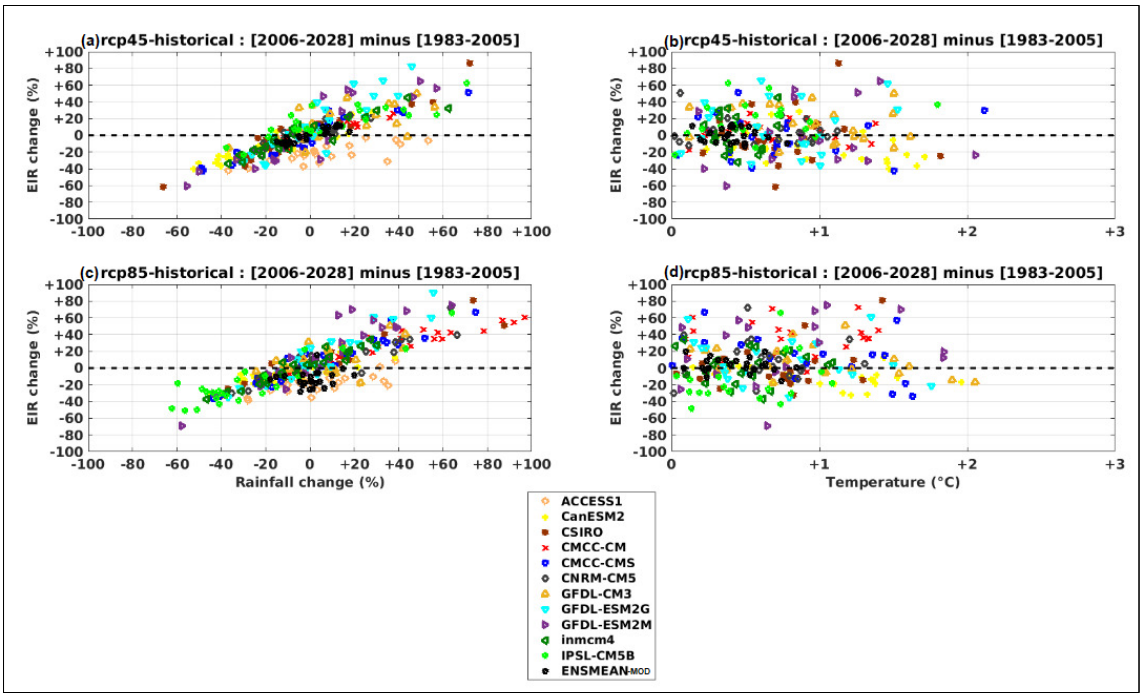

Figure S3: Relative changes in VECTRI-simulated malaria transmission versus percent precipitation change (left) and absolute mean surface temperature change (right) for RCP45 and RCP85 scenarios from bias-corrected CMIP5 GCM models for the near future: CanESM2, CSIRO, CMCC-CM, CMCC-CMS, CNRM-CM5, GFDL-CM3, GFDL-ESM2G, GFDL-ESM2M, inmcm4, IPSL-CM5B, ENSMEAN-MOD

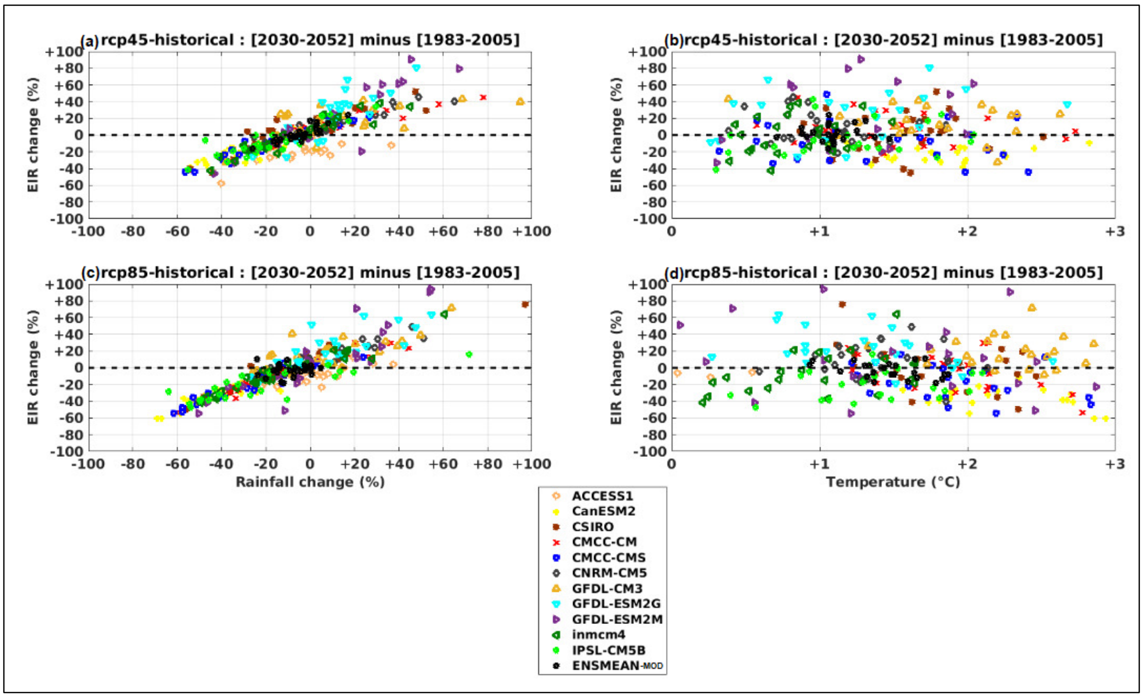

Figure S4: Relative changes in VECTRI-simulated malaria transmission versus percent precipitation change (left) and absolute mean surface temperature change (right) for RCP45 and RCP85 scenarios from bias-corrected CMIP5 GCM models for the middle future: CanESM2, CSIRO, CMCC-CM, CMCC-CMS, CNRM-CM5, GFDL-CM3, GFDL-ESM2G, GFDL-ESM2M, inmcm4, IPSL-CM5B, ENSMEAN-MOD

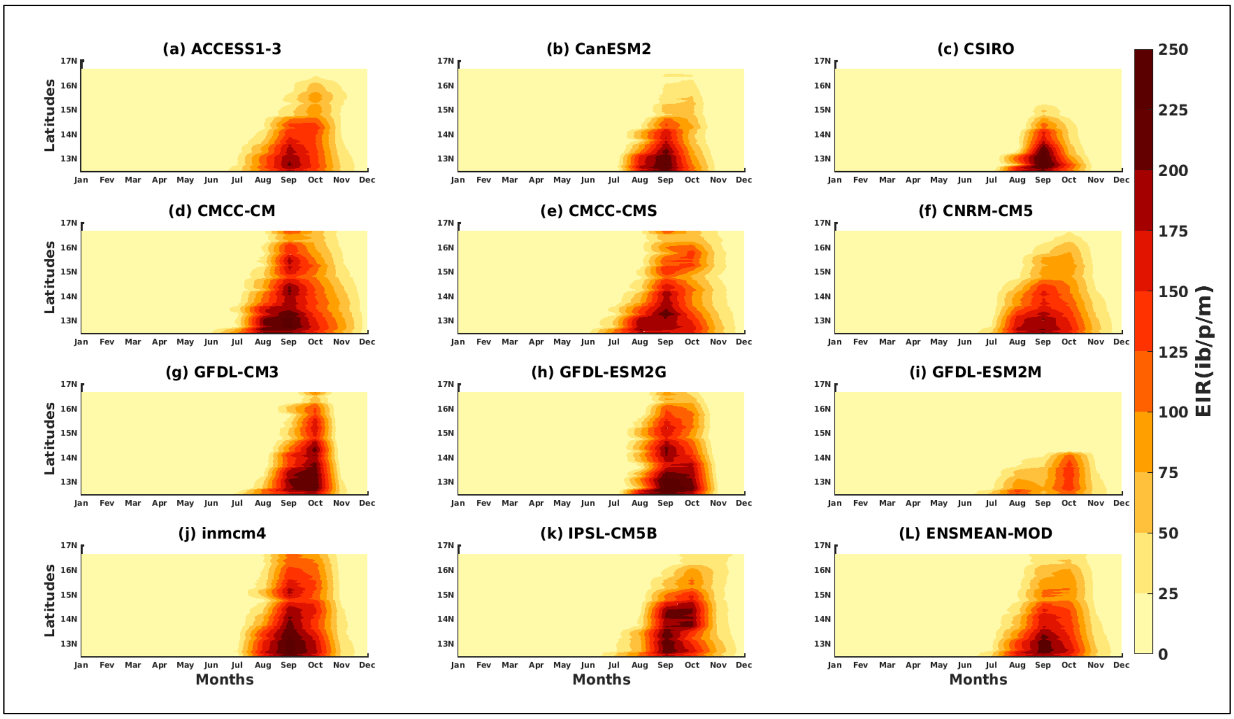

Figure S5: Hovmöller diagram of the annual EIR cycle of malaria in Senegal for the period 2006-2100: Simulations of the VECTRI model forced by rainfall and temperature bias-corrected CMIP5 GCM models: a) ACCESS1-3, b) CanESM2, c) CSIRO, d) CMCC-CM, e) CMCC-CMS, f) CNRM-CM5, g) GFDL-CM3, h) GFDL-ESM2G, i) GFDL-ESM2M, j) inmcm4, k) IPSL-CM5B, l) ENSMEAN-MOD for RCP4.5

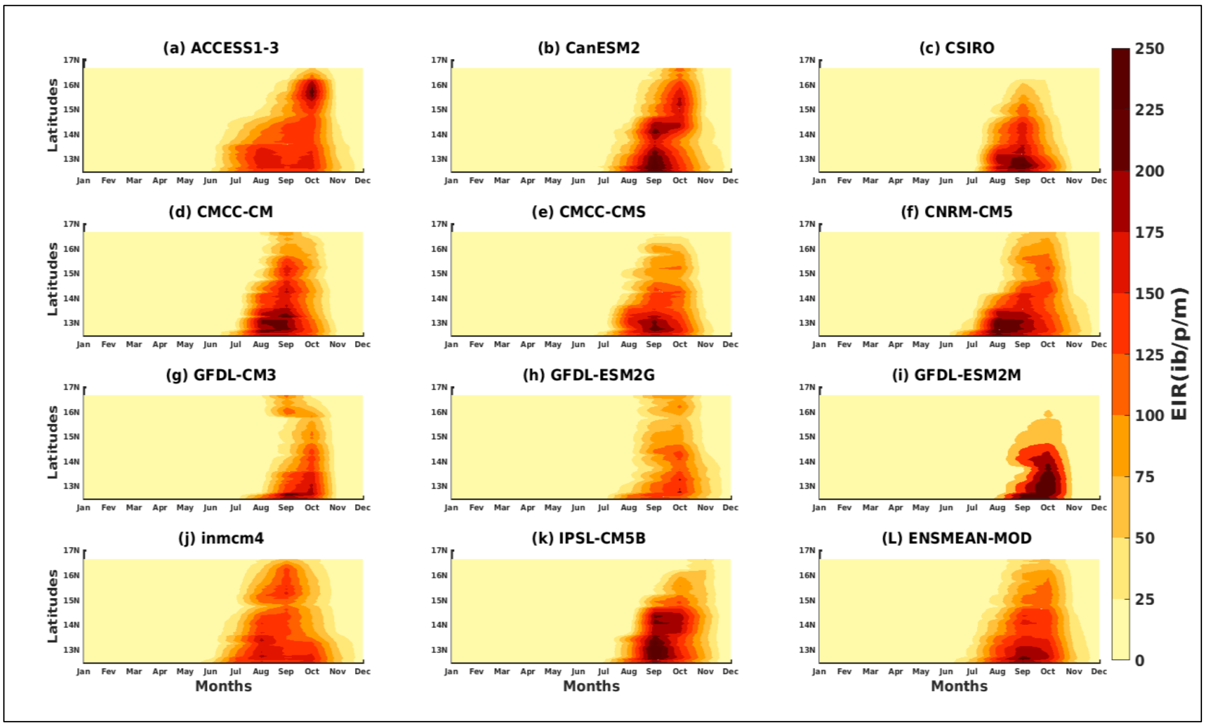

Figure S6: Hovmöller diagram of the annual EIR cycle of malaria in Senegal for the period 2006-2100: Simulations of the VECTRI model forced by rainfall and temperature bias-corrected CMIP5 GCM models: a)

ACCESS1-3, b) CanESM2, c) CSIRO, d) CMCC-CM, e) CMCC-CMS, f) CNRM-CM5, g) GFDL-CM3, h) GFDL-ESM2G, i) GFDL-ESM2M, j) inmcm4, k) IPSL-CM5B, l) ENSMEAN-MOD for RCP8.5

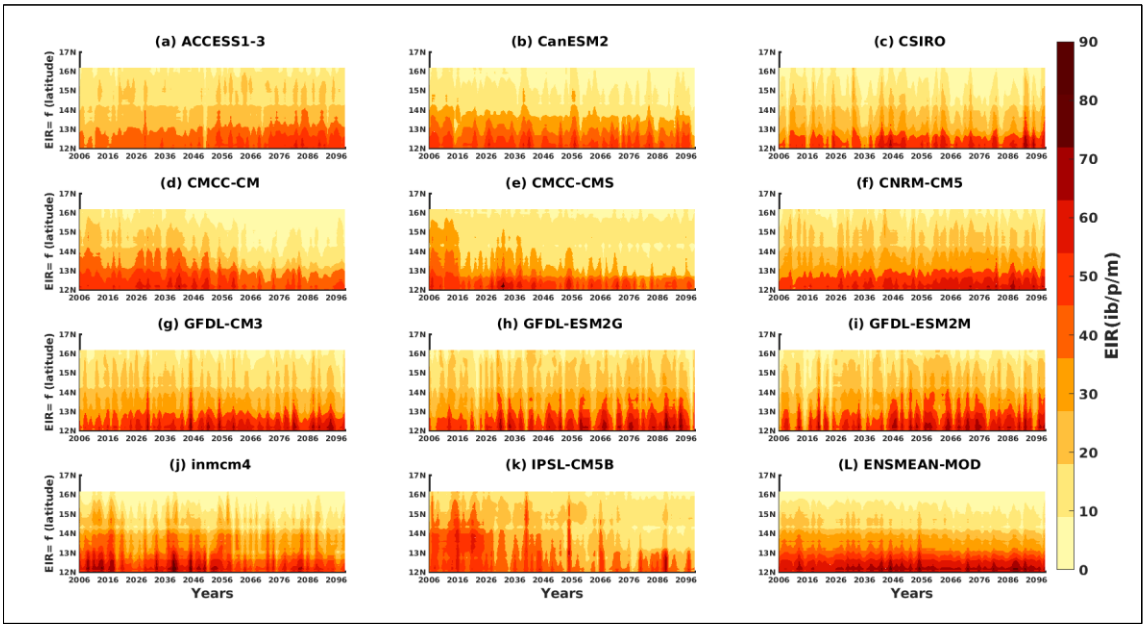

Figure S7: Hovmöller diagram of the interannual EIR cycle of malaria in Senegal for the period 2006-2100: Simulations of the VECTRI model forced by rainfall and temperature bias-corrected CMIP5 GCM models: a) ACCESS1-3, b) CanESM2, c) CSIRO, d) CMCC-CM, e) CMCC-CMS, f) CNRM-CM5, g) GFDL-CM3, h) GFDL-ESM2G, i) GFDL-ESM2M, j) inmcm4, k) IPSL-CM5B, l) ENSMEAN-MOD for RCP45

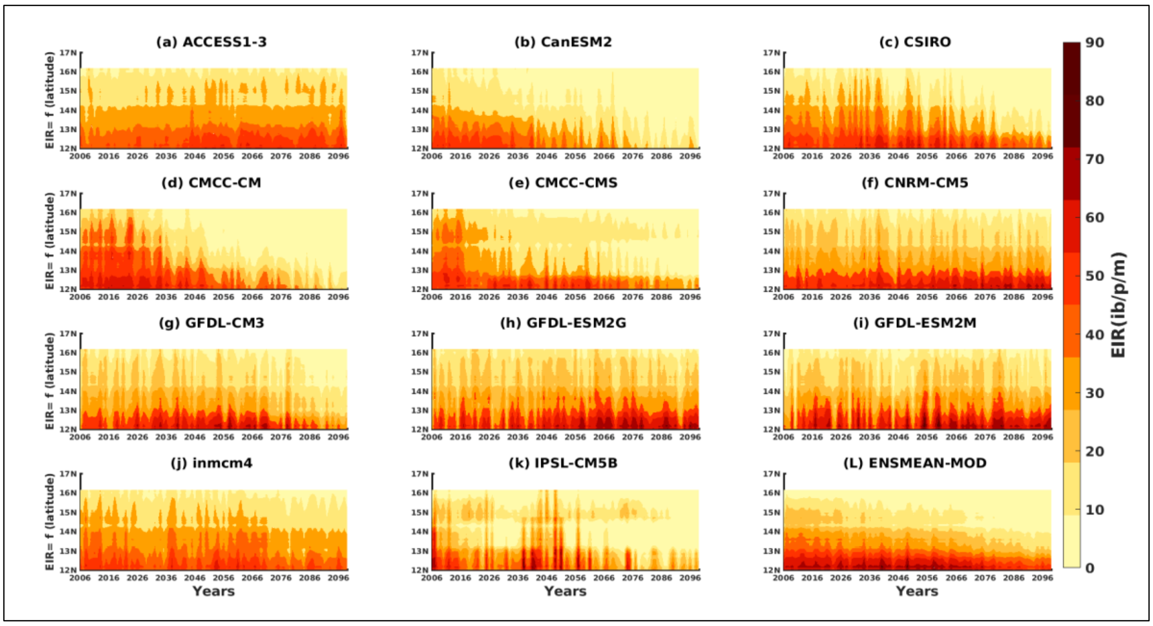

Figure S8: Hovmöller diagram of the interannual EIR cycle of malaria in Senegal for the period 1983-2005: Simulations of the VECTRI model forced by rainfall and temperature bias-corrected CMIP5 GCM models: a) ACCESS1-3, b) CanESM2, c) CSIRO, d) CMCC-CM, e) CMCC-CMS, f) CNRM-CM5, g) GFDL-CM3, h) GFDL-ESM2G, i) GFDL-ESM2M, j) inmcm4, k) IPSL-CM5B, l) ENSMEAN-MOD for RCP85.
